# Outcomes of a structured exercise and education program for hip and knee osteoarthritis across different clinical and geographic settings in Australia: A cohort study of 9,938 participants

**DOI:** 10.1016/j.ocarto.2026.100876

**Published:** 2026-09-07

**Authors:** Yoseph M. Alamneh, Dawn Aitken, Danilo De Oliveira Silva, Christian Barton, Laura Sutton, Joanne Kemp, Petr Otahal, Saliu Balogun, Marcella Ferraz Pazzinatto

**Affiliations:** a Menzies Institute for Medical Research, University of Tasmania, Hobart, Australia; b La Trobe University, Melbourne, Australia

**Keywords:** Osteoarthritis, Exercise, Outcomes, Location, Clinic settings

## Abstract

**Objective:**

In Australia, the Good Life with osteoArthritis from Denmark (GLA:D®) program is delivered across different settings. This study compared 3-month outcomes by clinic setting (public vs private), geographic location (rural/remote vs metropolitan), and education level among participants with hip and knee osteoarthritis.

**Design:**

This longitudinal registry-based cohort study used GLA:D® Australia registry data for participants enrolled between December 2017 and August 2025. Outcomes included pain, self-reported function, joint-and health-related quality of life, and physical function. Changes were estimated using mixed-effects linear regression.

**Results:**

Of 14,803 participants with baseline data, 9938 (67.1%) were included in complete-case analysis. About 74% attended metropolitan clinics, 84% attended private practices, and 44% had a university degree.

At 3-months, participants with knee osteoarthritis attending private clinics had greater improvements compared to those attending public clinics, including pain (worst pain mean difference = −4.8 [95% confidence interval = −6.4 to −3.2]), Knee injury and Osteoarthritis Outcome Score (KOOS-12) total (1.7 [0.7 to 2.6]) and KOOS-12 pain subscale scores (2.0 [1.0 to 3.1]). Outcomes did not differ between public and private clinics for hip osteoarthritis, nor by geographical location or education level for hip or knee osteoarthritis.

**Conclusions:**

Three-month improvements across all examined groups indicate that GLA:D® can deliver benefits in a wide range of settings, without statistically significant differences across geographic location or education level. Participants with knee osteoarthritis attending private clinics showed slightly better outcomes than those in public clinics, though the differences were small and likely not clinically meaningful.

## 1 Introduction

Osteoarthritis is a highly prevalent, painful, and disabling condition, and is among the leading causes of global disability [1]. In Australia, osteoarthritis affects over 2.2 million people and costs the healthcare system an estimated $AUD 4.3 billion, representing 2.9% of total health expenditure and 29% of spending on musculoskeletal conditions [2].

International clinical guidelines for managing osteoarthritis consistently recommend patient education, exercise, and weight management (where indicated) as first-line treatments for hip and knee osteoarthritis [[3], [4], [5], [6]]. Surgery is reserved for cases where non-surgical treatments have been unsuccessful [3,4,6]. Despite these recommendations, many people with osteoarthritis do not receive evidence-based first-line care and there is an over-reliance on medications and surgery to treat the condition [7].

Structured education and exercise programs, such as Good Life with osteoArthritis from Denmark (GLA:D®) [8], have been developed to facilitate delivery of guideline-recommended first-line care and demonstrated improvements in pain and function, and quality of life [[9], [10], [11], [12], [13]] in people with hip and knee osteoarthritis [8]. However, access to and delivery of evidence-based first-line care may vary according to healthcare setting, geographic location, and socioeconomic factors [[14], [15], [16], [17]]. Differences in service delivery between public and private healthcare, reduced access to services in rural and remote areas, and lower health literacy associated with lower educational attainment may influence participation and outcomes in osteoarthritis care [17]. These disparities contribute to poorer health outcomes, with Australians living in rural, remote, and socioeconomically disadvantaged areas experiencing higher rates of disability and premature mortality from chronic conditions, including musculoskeletal disorders, compared with those living in metropolitan areas [2,18].

In Australia, GLA:D® has been nationally implemented, in both private and public health settings across all states and territories. Prior evaluations of the program in Australia [19,20] indicate it is feasible and effective, with small to large improvements in outcome measures over 3-months. A previous nation-wide study [9] examined early implementation of GLA:D® in Australia, including clinician training, program adoption and outcomes among participants with knee osteoarthritis. The present study extends this work by using a substantially larger national registry cohort, including participants with hip and/or knee osteoarthritis. Additionally, we will specifically compare changes in outcomes between clinic setting, geographic location, and education level. Given GLA:D® is increasingly being implemented across different settings in different countries, including Australia, further investigation is needed to determine whether the program is consistently effective across different clinical and geographical settings.

This study compared 3-month outcomes for hip and knee osteoarthritis by clinic setting (public vs private), geographic location (metropolitan vs rural/remote), and educational level as indicator of socioeconomic status (SES). We hypothesised that participants attending private clinics, living in metropolitan areas, and with higher education levels would demonstrate better outcomes compared with those attending public clinics, living in rural or remote areas, and with lower education levels.

## 2 Methods

### 2.1 Design and setting

This longitudinal registry-based study used outcome data from the GLA:D® Australia registry and included patients enrolled from its inception in December 2017 to August 2025. GLA:D® registry data were collected using the Research Electronic Data Capture system hosted by La Trobe University. This report was prepared in line with the Strengthening the Reporting of Observational Studies in Epidemiology checklist [21] (eTable 1 in Supplementary file 1).

### 2.2 Patients and data collection

The dataset included 15,186 patients registered in the GLA:D® registry. Patients were eligible to participate in GLA:D® if they: (i) had clinically diagnosed hip and/or knee osteoarthritis [22] confirmed by GLA:D® clinician at enrolment; (ii) were aged 18 years and older; and (iii) were deemed appropriate for non-surgical management. Patients automatically received surveys via email at baseline and 3-months. The data included clinician-reported information on clinic setting and geographic location, and patient-reported outcome measures collected at baseline and 3-months. Details of the GLA:D® program and participant characteristics have been previously published [9]. Ethics approval for this research was provided by La Trobe University (HEC21303).

GLA:D® has three components, including: (i) two structured group education sessions (60–90 min each); (ii) 12 1-h group exercise classes run over a six-week period; and (iii) collection of outcome data in the GLA:D® Australia registry. Most patients are encouraged to complete the registry questionnaires online at home. In some settings, if patients expressed difficulty with the questionnaires at baseline, clinic staff supported them to complete the questionnaires, either online or using a paper-based version which was then uploaded by staff.

### 2.3 Outcomes

The following outcomes were evaluated at both baseline and 3-month follow-up. Outcomes were classified as joint-specific (for the affected hip or knee, including pain intensity) or non-joint-specific (overall health or physical performance). Performance-based tests were administered and supervised by GLA:D® trained clinicians using standardized protocols. Participants subsequently entered their results into the database. The tests included the 40-m walk test which measured walking speed (m/sec) (higher speed indicates better performance) and the sit-to-stand chair test which measured the number of repetitions completed in 30 s (higher values indicate better performance). Patient-reported outcomes included the 12-item Knee/Hip injury and Osteoarthritis Outcome Score (KOOS/HOOS-12), pain intensity over the past month, and health-related quality of life. The KOOS/HOOS-12 subscales (pain, function, and quality of life) were scored from 0 (worst pain/difficulty) to 100 (no pain/difficulty) [23]. Pain intensity was assessed using the Visual Analogue Scale (VAS) 0–100 mm, where 0 indicates no pain and 100 worst pain [24,25]. Health-related quality of life (mobility, self-care, usual activities, pain/discomfort, and anxiety/depression) was measured across European Quality of Life 5-Dimension 5-level (EQ-5D-5L), which ranges from 0 (worst health) to 1 (best health), while overall self-perceived health status was captured using the EQ-5D-5L VAS, which ranges from 0 (worst health) to 100 (best health) [26].

### 2.4 Geographical classifications

In Australia, several geographical classification systems are used to describe location and remoteness, including the Modified Monash Model (MMM). MMM classifications are based on the Australian Statistical Geography Standard-Remoteness Areas framework [27] and define whether a location is metropolitan, rural, remote, or very remote according to remoteness and population size. Clinics offering the GLA:D® program were categorised into metropolitan, rural, and remote zones, with MMM1 areas being classified as metropolitan (high population density and extensive infrastructure) and MMM 2 to 7 representing progressively more rural and remote locations, ranging from regional centres to large, medium, and small rural towns, and extending to remote and very remote communities [27]. For the purposes of this study, MMM categories 2 to 7 were combined into a single group labelled “rural/remote”.

### 2.5 Clinic settings

Australia has a mixed public and private healthcare system. Medicare is Australia's universal health insurance scheme and subsidises medial and hospital services. Public hospitals are jointly funded by the Australian federal, and state and territory governments, and eligible patients can receive treatment at no direct cost. Alternatively, patients may access healthcare through the private sector, which is funded through a combination of Medicare rebates, private health insurance, and/or out-of-pocket payments.

Clinics offering the GLA:D® program were classified into two categories: 1) private, including private hospitals and private clinics; 2) public, including public inpatients, public outpatients, and community clinics. Clinics providing care in aged care settings, sporting teams, or those recorded as unknown/other clinic setting were excluded from the analysis.

### 2.6 Education level

Education status is widely used in musculoskeletal research as a relatively stable and well-defined indicator of individual-level socioeconomic status [28]. Participants self-reported their highest achieved education level. For the purpose of analysis, we dichotomised education level into two groups: 1) no university degree, including primary, secondary, and post-secondary non-university qualifications such as diplomas; and 2) university degree, including undergraduate or postgraduate qualifications.

### 2.7 Statistical analysis

All patients with data from the baseline and 3-month assessment between December 2017 and August 2025 were included in the analyses. The baseline characteristics of the patients were reported as means with standard deviations for continuous variables and as number and proportions for categorical variables. We conducted complete case analysis, in those with complete data at baseline and 3-months for each outcome. Changes in outcomes at each time point were estimated using mixed-effects linear regression models, with subgroup analyses by clinic setting, geographic location, and education level. The mixed-effect model was chosen because it is robust to non-normality and accounts for correlated outcomes from repeated measures [29]. A total of 48 hypothesis tests were conducted across 16 outcomes and three subgroup comparisons (clinic setting, geographic location, and education level). Accordingly, a Bonferroni correction was applied [30], and statistical significance was defined as p < 0.001 (0.05 ÷ 48). No multivariable adjustment was undertaken because the study aimed to compare changes in outcomes across these subgroups rather than estimate independent associations. Clinically meaningful between-group differences were assessed by comparing the mean between-group difference in change and its 95% confidence interval against predefined thresholds: 10 points for the KOOS/HOOS [31] [32], 0.07 points for the EQ-5D-5L index [26], 4.83 points for the EQ-5D-5L VAS [33], 2 repetitions for the 30-s chair stand test [34], and 0.2 m/s for the 40-m walk test [34]. Baseline differences across clinic settings, geographic locations, and education levels were also evaluated. Sensitivity analyses were conducted to 1) compare characteristics between those included and excluded from our analyses; and 2) explore different cut-offs of education level as a measure of SES. As a sensitivity analysis, multiple imputation using chained equations was applied to account for missing data. The imputation model included age, gender, body mass index, education level, marital status, and baseline and 3-month outcome variables (further details are provided in eTable 2 in Supplementary file 1).

Analyses were conducted using Stata version 18 (StataCorp LLC), with a two-sided p-value <0.001 considered statistically significant, and results are presented with 95% confidence intervals.

## 3 Results

### 3.1 Participants

**Fig. 1:**
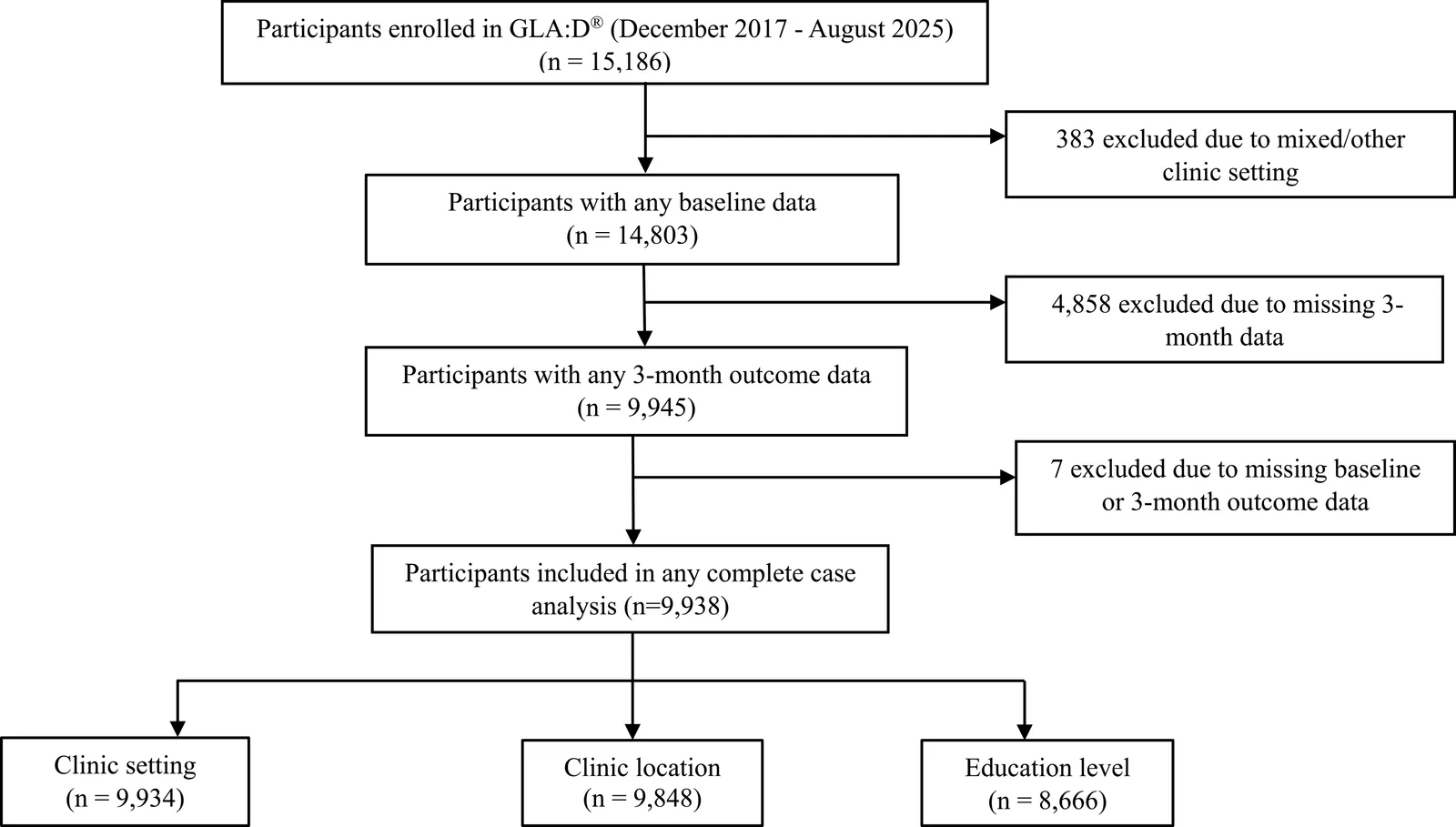
Flowchart of included participants.

Fig. 1 shows the included participants. Between December 2017 and August 2025, 15,186 participants enrolled in GLA:D®. Three hundred eighty-three participants were excluded due to mixed or non-standard clinic settings (including aged care, sporting teams, and unknown/other settings). Of the remaining 14,803 (97.5%) participants with baseline data, 9938 (67.1%) were included in the complete-case analysis, while 4865 (33.0%) were excluded due to missing baseline and/or 3-month outcome data required for the complete-case analysis.

### 3.2 Baseline characteristics

Table 1 presents baseline characteristics of participants who were included in the complete-case analysis. Mean age was 66.5 (SD 8.8) years, and mean BMI was 29.7 (SD 6.5) kg/m^2^, 72.2% were female, 80.3% reported that their primary joint affected was the knee. Most participants attended metropolitan clinics (74.4%), and private practices (83.5%).

**Table 1:** Demographic and baseline characteristics of participants included in complete-case analysis.

Baseline characteristics	Participants included in any complete-case analysis (n = 9938)
Age (years), mean (SD), n	66.5 (8.8), n = 9776
Body mass index (kg/m^2^), mean (SD), n	29.7 (6.5), n = 9809
**Sex, n (%)**	
Female	6711 (72.2)
Male	2586 (27.8)
**Marital status, n (%)**	
Single	502 (5.8)
Married/domestic partnership/de facto	6297 (72.7)
Widowed	736 (8.5)
Divorced	819 (9.5)
Separated	185 (2.1)
Prefer not to say	127 (1.5)
**Educational status, n (%)**	
No university degree	4825 (55.7)
University degree	3841 (44.3)
**Employment status, n (%)**	
Unemployed	206 (2.4)
Part-time	1394 (16.1)
Full-time	1560 (18.0)
Home duties	413 (4.8)
Student	10 (0.1)
Retired	5075 (58.6)
**Primary joint affected, n (%)**	
Knee	7977 (80.3)
Hip	1961 (19.7)
**State/territory, n (%)**	
Australian Capital Territory	969 (9.8)
New South Wales	828 (8.3)
Northern Territory	59 (0.6)
Queensland	866 (8.7)
South Australia	1419 (14.3)
Tasmania	470 (4.7)
Victoria	4376 (44.1)
Western Australia	947 (9.5)
**Clinic setting, n (%)**	
Private	8296 (83.5)
Public	1638 (16.5)
**Program location**^a^**, n (%)**	
Metropolitan	7331 (74.4)
Rural and remote	2517 (25.6)
**Knee measures, mean (SD), n**	
KOOS-12 total (0–100)^b^	50.9 (15.4), n = 7973
KOOS-12 pain (0–100)^b^	52.6 (15.5), n = 6971
KOOS-12 function (0–100)^b^	61.0 (18.7), n = 6965
KOOS-12 QoL (0–100)^b^	42.7 (17.0), n = 7968
Knee worst pain VAS (0–100)^c^	62.5 (23.3), n = 7955
Knee average pain VAS (0–100)^c^	44.4 (21.4), n = 7941
**Hip measures, mean (SD), n**	
HOOS-12 total (0–100)^b^	53.4 (15.5), n = 1960
HOOS-12 pain (0–100)^b^	52.7 (15.5), n = 1697
HOOS-12 function (0–100)^b^	63.8 (18.3), n = 1697
HOOS-12 QoL (0–100)^b^	46.9 (17.2), n = 1960
Hip worst pain VAS (0–100)^c^	60.3 (23.6), n = 1955
Hip average pain VAS (0–100)^c^	42.8 (21.3), n = 1954
**Combined measures**	
EQ-5D-5L, utility score (0–1)^d^	0.8 (0.1), n = 9885
EQ-5D-5L VAS (0–100)^e^	73.6 (18.4), n = 9879
30s chair stand test, count^f^	11.1 (4.0), n = 9738
40 m walk test, m/s^g^	1.5 (0.4), n = 8960

Abbreviations: EQ-5D-5L: European Quality of Life 5-Dimension 5-level; HOOS: Hip Disability and Osteoarthritis Outcome Score; KOOS: Knee Injury and Osteoarthritis Outcome Score; QoL: Quality of Life; n: Number; SD: Standard Deviation; VAS: Visual Analogue Scale.

a Remoteness measured using Modified Monash Model (MMM) categories MMM 1 to MMM 7. Areas classified as metropolitan correspond to MMM 1, which includes major cities characterized by high population densities and extensive infrastructure. Rural areas encompass MMM 2 to MMM 5, which include regional centres, large rural towns, medium rural towns, and small rural towns. Remote areas encompass MMM 6 and MMM 7, which consist of remote and very remote communities.

b Knee or hip-related pain, function, and quality of life are evaluated using KOOS/HOOS-12 question survey, scored from 0 (extreme difficulty/pain) to 100 (no difficulty/pain).

c Assessed average and worst pain scores for affected joints over the past month, using a VAS from 0 (no pain) to 100 (worst pain).

d QoL measured across five EQ-5D-5L dimensions, scores from 0 (worst health) to 1 (best health).

e QoL measured using VAS, scored 0 (worst health) to 100 (best health).

f Physiotherapists assessed physical function using the 30-s chair-stand - patients were asked to stand up and sit down from the chair to perform as many repetitions as possible in 30 s.

g Physiotherapists assessed physical function/walking speed using a 40-m walk test to measure the time (in seconds) taken to complete the 40-m walk.

eTable 3 outlines the characteristics for those included and excluded in this study.

Participants excluded from the analysis reported slightly worse hip/knee symptoms across numerous measures, and a lower proportion had a university degree, at baseline.

eTable 4 (Supplementary File 1) presents baseline characteristics of participants by clinic setting (public vs private). Participants with knee osteoarthritis attending private clinic reported less pain and better function compared to those attending public clinic (KOOS-12 total scores: mean difference [95% CI] 4.0 [3.1, 4.9], KOOS-12 pain: 3.5 [2.5, 4.5] and KOOS-12 function: 5.8 [4.7, 7.0]). Differences were also observed for hip measures, with less pain and better function among participants attending private clinics (HOOS-12 total: 2.9 [1.1, 4.8]; HOOS-12 pain: 2.8 [0.7, 4.8]; HOOS-12 function: 4.5 [2.1, 6.9]).

eTable 5 (Supplementary File 1) presents baseline characteristics of participants by geographical location (rural/remote vs metropolitan). Participants from metropolitan areas had comparable baseline scores to those from rural or remote areas across all 16 hip and knee measures.

eTable 6 (Supplementary file 1) presents baseline characteristics of participants by education level. Participants with a university degree reported better scores (i.e. less pain, better function and quality of life) at baseline across all 16 hip and knee measures compared with those without a university degree. For example, participants with a university degree reported higher KOOS-12 pain scores (i.e. lower pain): mean difference [95% CI] 5.6 [4.8, 6.3], lower VAS knee average pain: −7.5 [−8.5, −6.5], and higher HOOS-12 function scores: 3.9 [2.1, 5.6], indicating better hip function.

### 3.3 Three-month outcomes by clinic setting (public vs private), geographic location (metropolitan vs rural/remote) and education level

Table 2 describes the change in study outcomes between public and private clinics over 3- months. Participants attending both private and public clinic showed improvements across all 16 outcomes. Participants attending private clinics demonstrated statistically significant greater improvements in three knee-related outcomes: KOOS-12 total (mean difference 1.7 [95% CI: 0.7 to 2.6]), KOOS-12 pain (2.0 [1.0 to 3.1]), and VAS knee worst pain (−4.8 [−6.4 to −3.2]). No significant between-group differences were observed for hip-related outcomes or combined hip and knee outcomes.

**Table 2:** Change in study outcomes between public and private clinics over 3 months.

Study outcomes	Public, Mean (95% CI), n			Private, Mean (95% CI), n			Private vs public difference in change, mean (95% CI)^a^	P Value
	Baseline	3 months	Change at 3 months	Baseline	3 months	Change at 3 months		
**Knee measures**								
KOOS-12 total (0–100)^b^	47.6 (16.5), n = 1305	56.5 (18.4), n = 1305	8.9 (8.1, 9.8)	51.6 (15.1), n = 6585	62.1 (16.6), n = 6585	10.6 (10.2, 10.9)	1.7 (0.7, 2.6)	**<0.001**
KOOS-12 pain (0–100)^b^	49.7 (16.4), n = 1145	58.0 (18.9), n = 1145	8.2 (7.3, 9.2)	53.2 (15.3), n = 5746	63.5 (17.2), n = 5746	10.3 (9.8, 10.7)	2.0 (1.0, 3.1)	**<0.001**
KOOS-12 function (0–100)^b^	56.2 (19.4), n = 1143	64.6 (20.6), n = 1143	8.4 (7.3, 9.5)	62.0 (18.3), n = 5738	71.7 (18.6), n = 5738	9.7 (9.2, 10.2)	1.3 (0.1, 2.5)	0.038
KOOS-12 QoL (0–100)^b^	40.0 (18.2), n = 1302	49.4 (19.8), n = 1302	9.4 (8.4, 10.4)	43.2 (16.7), n = 6577	54.3 (18.1), n = 6577	11.1 (10.6, 11.5)	1.7 (0.6, 2.7)	0.002
Knee worst pain VAS (0–100)^c^	63.7 (22.9), n = 1294	51.9 (27.0), n = 1294	−11.8 (−13.3, −10.4)	62.2 (23.5), n = 6547	45.5 (26.7), n = 6547	−16.7 (−17.3, −16.0)	−4.8 (−6.4, −3.2)	<0.001
Knee average pain VAS (0–100)^c^	47.8 (22.0), n = 1292	37.8 (24.2), n = 1292	−10.0 (−11.3, −8.7)	43.6 (21.3), n = 6520	31.4 (22.5), n = 6520	−12.3 (−12.9, −11.7)	−2.3 (−3.7, −0.9)	0.002
**Hip measures**								
HOOS-12 total (0–100)^b^	51.0 (17.3), n = 301	57.7 (18.4), n = 301	6.8 (5.0, 8.5)	53.8 (15.1), n = 1629	61.3 (17.1), n = 1629	7.5 (6.8, 8.3)	0.8 (−1.2, 2.7)	0.440
HOOS-12 pain (0–100)^b^	50.4 (17.2), n = 258	58.0 (19.2), n = 258	7.7 (5.6, 9.8)	53.1 (15.1), n = 1411	61.3 (17.2), n = 1411	8.2 (7.3, 9.1)	0.5 (−1.8, 2.8)	0.664
HOOS-12 function (0–100)^b^	60.2 (20.3), n = 258	67.2 (20.2), n = 258	7.1 (4.7, 9.4)	64.4 (17.9), n = 1411	71.7 (18.7), n = 1411	7.3 (6.3, 8.3)	0.2 (−2.3, 2.8)	0.863
HOOS-12 QoL (0–100)^b^	45.5 (18.6), n = 301	51.6 (19.4), n = 301	6.1 (4.0, 8.1)	47.1 (16.9), n = 1629	54.3 (18.9), n = 1629	7.2 (6.3, 8.1)	1.1 (−1.1, 3.3)	0.324
Hip worst pain VAS (0–100)^c^	59.9 (24.4), n = 297	50.5 (26.1), n = 297	−9.3 (−12.4, −6.3)	60.4 (23.5), n = 1621	48.6 (27.2), n = 1621	−11.8 (−13.1, −10.5)	−2.5 (−5.8, 0.9)	0.149
Hip average pain VAS (0–100)^c^	44.9 (21.6), n = 298	37.0 (23.0), n = 298	−7.9 (−10.6, −5.2)	42.3 (21.2), n = 1618	33.4 (22.6), n = 1618	−9.0 (−10.1, −7.8)	−1.1 (−4.0, 1.9)	0.475
**Combined measures**								
EQ-5D-5L, utility score (0–1)^d^	0.72 (0.2), n = 1576	0.76 (0.2), n = 1576	0.04 (0.03, 0.05)	0.78 (0.1), n = 8153	0.82 (0.1), n = 8153	0.04 (0.04, 0.05)	0.00 (0.00, 0.00)	0.512
EQ-5D-5L VAS (0–100)^e^	69.8 (19.4), n = 1563	72.5 (19.9), n = 1563	2.6 (1.6, 3.7)	74.4 (18.0), n = 8150	76.9 (18.5), n = 8150	2.5 (2.0, 3.0)	−0.2 (−1.3, 1.0)	0.794
30s chair stand test, count^f^	10.3 (4.2), n = 903	13.2 (4.7), n = 903	2.9 (2.6, 3.1)	11.0 (3.6), n = 4422	14.2 (4.2), n = 4422	3.2 (3.0, 3.3)	0.3 (−0.1, 0.6)	0.063
40 m walk test, m/s^g^	1.38 (0.4), n = 812	1.57 (0.4), n = 812	0.18 (0.16, 0.21)	1.50 (0.4), n = 4132	1.69 (0.4), n = 4132	0.19 (0.18, 0.20)	0.01 (−0.02, 0.03)	0.603

Abbreviations: CI: Confidence Interval; EQ-5D-5L: European Quality of Life 5-Dimension 5-level; HOOS: Hip Disability and Osteoarthritis Outcome Score; KOOS: Knee Injury and Osteoarthritis Outcome Score; n: Number; QoL: Quality of Life; VAS: Visual Analogue Scale.

a The mean difference is calculated as private minus public. The change was estimated using linear mixed-effect model. No missing values were imputed.

b Knee or hip-related pain, function, and quality of life are evaluated using KOOS/HOOS-12 question survey, scored from 0 (extreme difficulty/pain) to 100 (no difficulty/pain).

c Assessed average and worst pain scores for affected joints over the past month, using a VAS from 0 (no pain) to 100 (worst pain).

d QoL measured across five EQ-5D-5L dimensions, scores from 0 (worst health) to 1 (best health).

e QoL measured using VAS, scored from 0 (worst health) to 100 (best health).

f Physiotherapists assessed physical function using the 30-s chair-stand - patients were asked to stand up and sit down from the chair to perform as many repetitions as possible in 30 s.

g Physiotherapists assessed physical function/walking speed using a 40-m walk test to measure the time (in seconds) taken to complete the 40-m walk.

Table 3 describes the change in study outcomes between metropolitan and rural and remote clinics over 3-months. Participants attending metropolitan and rural/remote clinics showed comparable improvements across all 16 hip and knee outcomes. For example, VAS knee average pain improved by −11.9 mm [95% confidence intervals -12.5 to −11.3] in metropolitan participants and −12.1 [−13.2 to −11.1] in rural/remote participants, with a non-significant between-group difference of 0.2 [−1.0 to 1.5]. No significant between-group differences were observed across any of the 16 outcomes.

**Table 3:** Change in study outcomes between metropolitan and rural/remote locations over 3 months.

Study outcomes	Metropolitan, Mean (95% CI), n			Rural and remote, Mean (95% CI), n			Metropolitan vs rural and remote difference in change, mean (95% CI)^a^	p value
	Baseline	3 months	Change at 3 months	Baseline	3 months	Change at 3 months		
**Knee measures**								
KOOS-12 total (0–100)^b^	51.1 (15.2), n = 5867	61.4 (16.8), n = 5867	10.3 (9.9, 10.7)	50.7 (16.0), n = 1946	61.0 (17.4), n = 1946	10.3 (9.6, 11.0)	0.0 (−0.8, 0.8)	0.992
KOOS-12 pain (0–100)^b^	53.0 (15.3), n = 5103	62.9 (17.3), n = 5103	9.9 (9.4, 10.3)	52.0 (16.0), n = 1718	62.2 (18.0), n = 1718	10.2 (9.4, 11.0)	−0.3 (−1.3, 0.6)	0.457
KOOS-12 function (0–100)^b^	61.4 (18.5), n = 5096	70.9 (18.9), n = 5096	9.5 (9.0, 10.0)	60.2 (19.1), n = 1715	69.8 (19.5), n = 1715	9.6 (8.7, 10.5)	−0.1 (−1.1, 1.0)	0.922
KOOS-12 QoL (0–100)^b^	42.6 (16.7), n = 5860	53.6 (18.2), n = 5860	10.9 (10.5, 11.4)	43.0 (17.5), n = 1942	53.5 (19.0), n = 1942	10.5 (9.7, 11.3)	0.4 (−0.5, 1.4)	0.337
Knee worst pain VAS (0–100)^c^	62.3 (23.1), n = 5835	46.4 (26.6), n = 5835	−15.9 (−16.6, −15.2)	62.6 (24.2), n = 1928	46.7 (27.4), n = 1928	−15.9 (−17.1, −14.7)	0.0 (−1.4, 1.3)	0.957
Knee average pain VAS (0–100)^c^	44.0 (21.2), n = 5805	32.2 (22.7), n = 5805	−11.9 (−12.5, −11.3)	44.9 (22.0), n = 1929	32.7 (23.4), n = 1929	−12.1 (−13.2, −11.1)	0.2 (−1.0, 1.5)	0.689
**Hip measures**								
HOOS-12 total (0–100)^b^	53.6 (15.4), n = 1388	60.8 (17.2), n = 1388	7.2 (6.4, 8.0)	52.8 (15.8), n = 538	60.6 (17.6), n = 538	7.8 (6.5, 9.2)	−0.7 (−2.2, 0.9)	0.417
HOOS-12 pain (0–100)^b^	53.1 (15.3), n = 1196	60.9 (17.5), n = 1196	7.8 (6.8, 8.8)	51.6 (15.8), n = 469	60.3 (17.6), n = 469	8.**7** (7.1, 10.2)	−0.9 (−2.7, 0.9)	0.336
HOOS-12 function (0–100)^b^	64.1 (18.4), n = 1196	71.3 (18.9), n = 1196	7.2 (6.1, 8.3)	62.9 (18.5), n = 469	70.1 (19.3), n = 469	7.2 (5.5, 8.9)	0.0 (−2.0, 2.1)	0.979
HOOS-12 QoL (0–100)^b^	47.0 (16.9), n = 1388	53.8 (18.7), n = 1388	6.8 (5.9, 7.8)	46.4 (17.9), n = 538	53.8 (19.7), n = 538	7.4 (5.9, 9.0)	−0.6 (−2.4, 1.2)	0.498
Hip worst pain VAS (0–100)^c^	60.3 (23.3), n = 1383	48.7 (26.9), n = 1383	−11.6 (−13.0, −10.2)	60.3 (24.5), n = 531	49.5 (27.1), n = 531	−10.8 (−13.1, −8.5)	−0.8 (−3.5, 1.9)	0.563
Hip average pain VAS (0–100)^c^	42.5 (21.2), n = 1380	33.4 (22.6), n = 1380	−9.1 (−10.4, −7.8)	43.3 (21.3), n = 532	35.4 (22.6), n = 532	−7.9 (−9.9, −5.9)	−1.2 (−3.6, 1.2)	0.328
**Combined measures**								
EQ-5D-5L, utility score (0–1)^d^	0.77 (0.1), n = 7196	0.81 (0.1), n = 7196	0.04 (0.04, 0.05)	0.76 (0.1), n = 2453	0.81 (0.1), n = 2453	0.04 (0.04, 0.05)	0.00 (−0.01, 0.01)	0.967
EQ-5D-5L VAS (0–100)^e^	73.7 (18.2), n = 7184	76.2 (18.7), n = 7184	2.5 (2.0, 3.0)	73.7 (18.8), n = 2451	76.4 (19.0), n = 2451	2.7 (1.8, 3.5)	−0.2 (−1.2, 0.8)	0.729
30s chair stand test, count^f^	10.9 (3.7), n = 3943	14.0 (4.3), n = 3943	3.1 (3.0, 3.2)	11.0 (3.9), n = 1345	14.0 (4.3), n = 1345	3.1 (2.8, 3.3)	0.1 (−0.2, 0.3)	0.602
40 m walk test, m/s^g^	1.48 (0.4), n = 3681	1.68 (0.4), n = 3681	0.19 (0.18, 0.21)	1.47 (0.4), n = 1231	1.65 (0.4), n = 1231	0.18 (0.16, 0.20)	0.02 (−0.01, 0.04)	0.146

Abbreviations: CI: Confidence Interval; EQ-5D-5L: 5-Level: Euroqol-5-Dimensional Version; HOOS: Hip Disability and Osteoarthritis Outcome Score; KOOS: Knee Injury and Osteoarthritis Outcome Score; n: Number; QoL: Quality of Life; VAS: Visual Analogue Scale.

a The mean difference calculated as metropolitan minus rural/remote. The change was estimated using linear mixed-effect model. No missing values were imputed.

b Knee or hip-related pain, function, and quality of life are evaluated using KOOS/HOOS-12 question survey, scored from 0 (extreme difficulty/pain) to 100 (no difficulty/pain).

c Assessed average and worst pain scores for affected joints over the past month, using a VAS from 0 (no pain) to 100 (worst pain).

d QoL measured across five EQ-5D-5L dimensions, scores from 0 (worst health) to 1 (best health).

e QoL measured using VAS, scored 0 (worst health) to 100 (best health).

f Physiotherapists assessed physical function using the 30-s chair-stand - patients were asked to stand up and sit down from the chair to perform as many repetitions as possible in 30 s.

g Physiotherapists assessed physical function/walking speed using a 40-m walk test to measure the time (in seconds) taken to complete the 40-m walk.

Table 4 describes the change in study outcomes by education level over 3-months. Both participants with a university degree and those without a university degree showed improvements across all 16 outcomes, with no significant between-group differences. These findings were consistent when different cut-offs of education level were used (data not shown).

**Table 4:** Change in study outcomes based on highest level of education over 3 months.

Study outcomes	No university degree			University degree			University degree vs no university degree in change mean (95% CI)^a^	P Value
	Baseline, Mean (SD), n	3 months, Mean (SD), n	Change at 3 months, Mean (95% CI), n	Baseline, Mean (SD), n	3 months, Mean (SD), n	Change at 3 months, Mean (95% CI), n		
**Knee measures**								
KOOS-12 total (0–100)^b^	49.9 (15.3), n = 3834	59.9 (17.5), n = 3834	10.0 (9.5, 10.5)	54.9 (14.4), n = 3056	64.9 (15.6), n = 3056	10.0 (9.5, 10.5)	0.0 (−0.7, 0.7)	0.975
KOOS-12 pain (0–100)^b^	50.2 (15.5), n = 3832	60.2 (18.0), n = 3832	10.0 (9.5, 10.6)	55.8 (14.9), n = 3056	65.6 (16.6), n = 3056	9.8 (9.2, 10.4)	−0.2 (−1.0, 0.6)	0.607
KOOS-12 function (0–100)^b^	58.1 (18.8), n = 3828	67.8 (19.4), n = 3828	9.7 (9.0, 10.3)	64.6 (17.8), n = 3051	73.9 (17.8), n = 3051	9.3 (8.6, 10.0)	−0.4 (−1.3, 0.5)	0.412
KOOS-12 QoL (0–100)^b^	41.3 (17.7), n = 3829	51.7 (19.4), n = 3829	10.3 (9.8, 10.9)	44.4 (16.5), n = 3051	55.3 (17.5), n = 3051	10.9 (10.3, 11.5)	0.6 (−0.3, 1.4)	0.200
Knee worst pain VAS (0–100)^c^	65.0 (22.6), n = 3810	50.2 (26.7), n = 3810	−14.9 (−15.7, −14.0)	60.0 (23.7), n = 3042	44.3 (26.2), n = 3042	−15.7 (−16.6, −14.7)	−0.8 (−2.1, 0.5)	0.221
Knee average pain VAS (0–100)^c^	48.1 (21.4), n = 3794	36.7 (23.5), n = 3794	−11.4 (−12.1, −10.6)	40.5 (21.0), n = 3033	29.0 (21.6), n = 3033	−11.6 (−12.4, −10.8)	−0.2 (−1.3, 0.9)	0.721
**Hip measures**								
HOOS-12 total (0–100)^b^	53.6 (15.5), n = 924	61.2 (17.3), n = 924	7.7 (6.7, 8.7)	55.6 (14.7), n = 745	62.7 (16.3), n = 745	7.2 (6.0, 8.3)	−0.5 (−2.0, 1.0)	0.509
HOOS-12 pain (0–100)^b^	51.6 (15.7), n = 924	60.1 (17.9), n = 924	8.5 (7.4, 9.6)	54.0 (15.0), n = 745	61.6 (17.1), n = 745	7.6 (6.3, 8.8)	−1.0 (−2.6, 0.7)	0.255
HOOS-12 function (0–100)^b^	62.1 (18.6), n = 924	69.7 (19.7), n = 924	**7.7** (6.4, 8.9)	65.9 (17.9), n = 745	72.6 (18.1), n = 745	6.7 (5.4, 8.1)	−0.9 (−2.8, 0.9)	0.320
HOOS-12 QoL (0–100)^b^	47.0 (17.8), n = 924	53.8 (19.4), n = 924	**6.8 (5.6, 8.0)**	46.9 (16.8), n = 745	54.1 (18.7), n = 745	**7.2 (5.9, 8.5)**	0.4 (−1.4, 2.1)	0.678
Hip worst pain VAS (0–100)^c^	61.0 (23.2), n = 917	50.2 (26.5), n = 917	−10.8 (−12.5, −9.1)	60.3 (24.0), n = 741	49.2 (26.7), n = 741	−11.1 (−13.0, −9.1)	−0.3 (−2.9, 2.3)	0.845
Hip average pain VAS (0–100)^c^	45.0 (21.2), n = 917	36.2 (22.6), n = 917	−8.7 (−10.3, −7.2)	40.8 (21.0), n = 740	32.7 (22.2), n = 740	−8.1 (−9.8, −6.4)	0.6 (−1.7, 2.9)	0.591
**Combined measures**								
EQ-5D-5L, utility score (0–1)^d^	0.76 (0.2), n = 4718	0.80 (0.2), n = 4718	0.04 (0.04, 0.05)	0.78 (0.1), n = 3777	0.82 (0.1), n = 3777	0.04 (0.04, 0.05)	0.00 (−0.01, 0.01)	0.829
EQ-5D-5L VAS (0–100)^e^	73.6 (19.3), n = 4713	75.6 (19.3), n = 4713	2.1 (1.5, 2.7)	74.0 (17.9), n = 3776	76.5 (18.3), n = 3776	2.5 (1.8, 3.2)	0.4 (−0.5, 1.4)	0.340
30s chair stand test, count^f^	10.6 (3.8), n = 2355	13.8 (4.4), n = 2355	3.1 (3.0, 3.3)	11.4 (3.9), n = 1933	14.5 (4.5), n = 1933	3.1 (2.9, 3.2)	−0.1 (−0.3, 0.2)	0.577
40 m walk test, m/s^g^	1.41 (0.4), n = 2151	1.60 (0.4), n = 2151	0.19 (0.17, 0.20)	1.53 (0.4), n = 1792	1.72 (0.4), n = 1792	0.19 (0.18, 0.21)	0.01 (−0.02, 0.03)	0.544

Abbreviations: CI: Confidence Interval; EQ-5D-5L: European Quality of Life 5-Dimension 5-level; HOOS: Hip Disability and Osteoarthritis Outcome Score; KOOS: Knee Injury and Osteoarthritis Outcome Score; n: Number; QoL: Quality of Life; VAS: Visual Analogue Scale.

a The mean difference is calculated as university degree minus no university degree. The change was estimated using linear mixed-effect model. No missing values were imputed.

b Knee or hip-related pain, function, and quality of life are evaluated using KOOS/HOOS-12 question survey, scored from 0 (extreme difficulty/pain) to 100 (no difficulty/pain).

c Assessed average and worst pain scores for affected joints over the past month, using a VAS from 0 (no pain) to 100 (worst pain).

d QoL measured across five EQ-5D-5L dimensions, scores from 0 (worst health) to 1 (best health).

e QoL measured using VAS, scored 0 (worst health) to 100 (best health).

f Physiotherapists assessed physical function using the 30-s chair-stand - patients were asked to stand up and sit down from the chair to perform as many repetitions as possible in 30 s.

g Physiotherapists assessed physical function/walking speed using a 40-m walk test to measure the time (in seconds) taken to complete the 40-m walk.

Results incorporating multiple imputation (eTable 7-9 in Supplementary file 1) produced no meaningful change in interpretation compared with the presented complete-case analysis.

## 4 Discussion

This study demonstrated that people with hip and knee osteoarthritis experienced clinically meaningful short-term improvements in pain, physical function, and quality of life following participation in the GLA:D® program. These benefits were largely consistent across clinic settings (public vs private), geographic locations (metropolitan vs rural/remote), and educational levels, supporting the program's effectiveness irrespective of socioeconomic or geographic position. While there were some baseline differences observed between participants with hip and knee osteoarthritis from public clinics and those with lower education levels, their improvements at 3-months were largely comparable. One exception was observed in knee-specific outcomes, where participants from private clinics reported slightly greater improvements than those from public clinics.

In Australia, well-documented health disparities exist, with individuals living in rural or remote areas and those with lower socioeconomic position often experiencing poorer outcomes for chronic conditions [16]. Our findings showed no meaningful differences in short-term outcomes by geographic location or education level, and only slightly greater improvements for knee osteoarthritis participants attending private clinics. These results are consistent with previous evaluations in Denmark [8], Canada [35], and Australia [9], which demonstrate improvements in pain, function, and quality of life following participation in the GLA:D® program, irrespective of socioeconomic or geographic position. This may reflect the program's standardised delivery, including consistent content, dosage, and structure. Together, these findings suggest that structured education and exercise programs for hip and knee osteoarthritis can be implemented across different settings, without loss of effectiveness, supporting its wider implementation to improve access to evidence-based osteoarthritis care. Participants with knee osteoarthritis attending private clinics demonstrated slightly greater improvements than those attending public clinics and several factors may explain this. Service delivery may differ between public and private, with greater appointment flexibility and lower wait times in private settings [36,37]. In addition, private services operate on a fee-for-service basis, which may influence patient expectations, motivation, adherence and perceived improvement [38] [39]. The differences could also be that private clinics typically serve people with higher SES, which is also known to be associated with better health outcomes [16]. However, it is important to note that the differences seen between public and private in this study were small and unlikely to be clinically meaningful.

Education level was not associated with differences in short-term outcomes, suggesting similar program benefits across socioeconomic groups. Education was used as a proxy for individual SES because it tends to remain stable across adulthood, is less affected by disease onset, is less prone to non-response bias than income [40], and provides an individual-level measure compared with area-based measures such as the Socio-Economic Indexes for Areas [41]. Consistent with prior evidence [42], less educated participants reported higher pain and poorer function at baseline compared to those who were more educated. Despite these baseline differences, improvements in pain, quality of life, and physical function following the GLA:D® program were comparable across education levels. This suggests that structured, supervised education and exercise programs may deliver similar short-term benefits across socioeconomic groups.

Our findings suggest that program benefits are consistent across different funding models and access to care, which is reassuring. Healthcare in Australia is constrained within the public and private funding model. In public clinics, the program is fully subsidised, reducing cost barriers to participation, whereas delivery in private clinics requires a patient out-of-pocket payment, which can limit access among lower socioeconomic groups. However, SES is multidimensional, and education alone may not fully capture socioeconomic position. Future research incorporating multiple SES measures may help clarify how socioeconomic factors influence program engagement and outcomes and inform strategies to optimise equitable delivery.

While this study provides insights for broader implementation of the GLA:D® program and its benefits for hip and knee osteoarthritis across clinic setting, geographic location, and educational level, there are limitations that should be considered when interpreting the findings. First, this was a registry-based, observational study without a control group and improvements cannot be attributed to program participation alone. It is possible that factors other than the program influenced the observed improvements, including regression to the mean, contextual factors, and placebo effects [43]. Second, the proportion of missing data across all outcomes was substantial. Multiple imputation was used to address this and showed similar results between the imputed and complete-case analyses. However, this method assumes the data were missing at random [44] and there is a risk that missing data was related to unobserved factors [44]. Therefore, although the sensitivity analyses showed similar results, the substantial level of missing data limits confidence in the magnitude of the observed effects, and the findings need replication in additional studies. Third, although performance-based tests were administered and supervised by GLA:D® trained clinicians using standardized protocols, the execution of the tests and accuracy of the results as entered by participants could not be verified. Fourth, education level was used as a proxy for SES and may not fully capture socioeconomic position. The education cut-offs we used to dichotomise the data were arbitrary; however, using different cut offs produced the same findings. Lastly, some subgroup analyses included relatively small numbers of participants, particularly in rural/remote and public clinic settings. For example, the public clinic subgroup included 258 participants for the HOOS-12 pain outcome. Although no clinically meaningful between-group differences were observed, the small subgroup sizes may have reduced statistical power to detect smaller effects. Furthermore, no multivariable adjustment was performed to estimate independent effects of clinic setting, geographic location, or education level.

The major strengths of this study include the use of real-world registry data, inclusion of participants from metropolitan and rural/remote areas across all Australian states and territories, and a large, diverse sample with standardised group-based delivery by certified clinicians across varied settings. These factors enhance the generalisability of the findings to routine clinical delivery of structured education and exercise program for osteoarthritis.

## 5 Conclusion

Three-month improvements across all examined groups indicate that GLA:D® can deliver benefits in a wide range of settings, without statistically significant differences across geographic location or education level. Participants with knee osteoarthritis attending private clinics showed slightly better outcomes than those in public clinics, though the differences were small and likely not clinically meaningful.

## Author contributions

All authors contributed to the drafting and revision of the manuscript and approved the final version. Yoseph Alamneh (yosephmerkeb.alamneh@utas.edu.au) ensures the integrity of the entire work.

**Conception and design:** Dawn Aitken, Christian Barton, Yoseph Alamneh, Laura Sutton, Saliu Balogun, Danilo De Oliveira Silva, Marcella Ferraz Pazzinatto, Joanne Kemp.

**Acquisition of data:** Yoseph Alamneh, Laura Sutton, Christian Barton, Saliu Balogun, Danilo De Oliveira Silva, Marcella Ferraz Pazzinatto, Joanne Kemp, Dawn Aitken.

**Analysis:** Yoseph Alamneh, Laura Sutton, Dawn Aitken, Petr Otahal.

**Interpretation of the data:** All authors.

**Drafting of the manuscript:** Yoseph Alamneh, Laura Sutton, Dawn Aitken.

**Critical review of the manuscript for important intellectual content:** All authors.

## Data availability statement

The data can be available from the corresponding author on reasonable request, subject to a data sharing agreement.

## Declaration of generative AI

Generative AI and AI-assisted technologies were NOT used in the preparation of this work.

## Funding

No specific funding was received for this work.

## Declaration of competing interest

Yoseph Alamneh: Nothing to disclose.

Dawn Aitken: Nothing to disclose.

Danilo De Oliveira Silva: Nothing to disclose.

Christian Barton: Nothing to disclose.

Laura Sutton: Nothing to disclose.

Saliu Balogun: Nothing to disclose.

Joanne Kemp: Nothing to disclose.

Petr Otahal: Nothing to disclose.

Marcella Ferraz Pazzinatto: MFP is the GLA:D® Australia project manager, part of her salary is funded by the program.
