# Supplementary material for: Outcomes of a structured exercise and education program for hip and knee osteoarthritis across different clinical and geographic settings in Australia: A cohort study of 9,938 participants
Source: Osteoarthr Cartil Open. 2026 Sep 7;8(4):100876. doi: 10.1016/j.ocarto.2026.100876 (PMC13583589; doi:10.1016/j.ocarto.2026.100876)
Supplement: Supplementary file 1 [file mmc1.docx]

**Supplementary File 1**

**eTable 1**. STrengthening the Reporting of OBservational studies in Epidemiology (STROBE) Checklist for Cohort Studies

|  | Item No | Recommendation | Reported on page/ Y/N/A |
| --- | --- | --- | --- |
| **Title and abstract** | 1 | (*a*) Indicate the study’s design with a commonly used term in the title or the abstract | 1 |
|  |  | (*b*) Provide in the abstract an informative and balanced summary of what was done and what was found | 1-2 |
| Introduction | | | |
| Background/rationale | 2 | Explain the scientific background and rationale for the investigation being reported | 3 |
| Objectives | 3 | State specific objectives, including any prespecified hypotheses | 4 |
| Methods | | | |
| Study design | 4 | Present key elements of study design early in the paper | 4 |
| Setting | 5 | Describe the setting, locations, and relevant dates, including periods of recruitment, exposure, follow-up, and data collection | 4 |
| Participants | 6 | (*a*) Give the eligibility criteria, and the sources and methods of selection of participants. Describe methods of follow-up | 4 |
|  |  | (*b*) For matched studies, give matching criteria and number of exposed and unexposed | N/A |
| Variables | 7 | Clearly define all outcomes, exposures, predictors, potential confounders, and effect modifiers. Give diagnostic criteria, if applicable | 5 |
| Data sources/ measurement | 8 | For each variable of interest, give sources of data and details of methods of assessment (measurement). Describe comparability of assessment methods if there is more than one group | 5 |
| Bias | 9 | Describe any efforts to address potential sources of bias | N/A |
| Study size | 10 | Explain how the study size was arrived at | N/A |
| Quantitative variables | 11 | Explain how quantitative variables were handled in the analyses. If applicable, describe which groupings were chosen and why | N/A |
| Statistical methods | 12 | (*a*) Describe all statistical methods, including those used to control for confounding | 7 |
|  |  | (*b*) Describe any methods used to examine subgroups and interactions | N/A |
|  |  | (*c*) Explain how missing data were addressed | 7 |
|  |  | (*d*) If applicable, explain how loss to follow-up was addressed | N/A |
|  |  | (*e*) Describe any sensitivity analyses | 7 |
| Results | | | |
| Participants | 13 | (a) Report numbers of individuals at each stage of study—eg numbers potentially eligible, examined for eligibility, confirmed eligible, included in the study, completing follow-up, and analysed | 7 |
|  |  | (b) Give reasons for non-participation at each stage | N/A |
|  |  | (c) Consider use of a flow diagram | Y |
| Descriptive data | 14 | (a) Give characteristics of study participants (eg demographic, clinical, social) and information on exposures and potential confounders | 8 |
|  |  | (b) Indicate number of participants with missing data for each variable of interest | Y |
|  |  | (c) Summarise follow-up time (eg, average and total amount) | N/A |
| Outcome data | 15 | Report numbers of outcome events or summary measures over time | N/A |
| Main results | 16 | (*a*) Give unadjusted estimates and, if applicable, confounder-adjusted estimates and their precision (eg, 95% confidence interval). Make clear which confounders were adjusted for and why they were included | Y |
|  |  | (*b*) Report category boundaries when continuous variables were categorized | Y |
|  |  | (*c*) If relevant, consider translating estimates of relative risk into absolute risk for a meaningful time period | N/A |
| Other analyses | 17 | Report other analyses done—eg analyses of subgroups and interactions, and sensitivity analyses | 7 |
| Discussion | | | |
| Key results | 18 | Summarise key results with reference to study objectives | 10 |
| Limitations | 19 | Discuss limitations of the study, taking into account sources of potential bias or imprecision. Discuss both direction and magnitude of any potential bias | 12 |
| Interpretation | 20 | Give a cautious overall interpretation of results considering objectives, limitations, multiplicity of analyses, results from similar studies, and other relevant evidence | 12 |
| Generalisability | 21 | Discuss the generalisability (external validity) of the study results | 12 |
| Other information | | | |
| Funding | 22 | Give the source of funding and the role of the funders for the present study and, if applicable, for the original study on which the present article is based | N/A |

**eTable 2.** Additional detailed description regarding multiple imputations.

| Information | Handling |
| --- | --- |
| Software | Stata (version 18; StataCorp, College Station,  TX, USA) |
| Method | Fully conditional specification |
| Seed | A positive integer was defined to start the  pseudo-random number generator (SEED) to be  able to regenerate results |
| Number of imputations | 50 imputed datasets were created |
| Variables included in each of the model | Age, body mass index, gender, education level, marital status, and baseline and 3-month outcome variables |

**eTable 3**. Demographic and baseline characteristics of participants included and excluded in this study.

| Baseline Characteristics | Participants included in any complete-case analysis below (n = 9,938) | Participants excluded due to missing data in any complete-case analysis below (n = 4,865) | Mean difference for continuous measures (95% CI) |
| --- | --- | --- | --- |
| Age (years), mean (SD), n | 66.5 (8.8), n = 9,776 | 64.9 (9.8), n = 4,630 | 1.6 (1.3, 1.9) |
| Body mass index (kg/m^2^), mean (SD), n | 29.7 (6.5), n = 9,809 | 30.6 (6.7), n = 4,642 | -0.9 (-1.1, -0.6) |
| **Sex, n (%)** |  |  |  |
| Female | 6,711 (72.2) | 3,000 (70.2) |  |
| Male | 2,586 (27.8) | 1,271 (29.8) |  |
| **Marital Status, n (%)** |  |  |  |
| Single | 502 (5.8) | 202 (5.3) |  |
| Married/domestic partnership/de facto | 6,297 (72.7) | 2,678 (70.6) |  |
| Widowed | 736 (8.5) | 334 (8.8) |  |
| Divorced | 819 (9.5) | 401 (10.6) |  |
| Separated | 185 (2.1) | 96 (2.5) |  |
| Prefer not to say | 127 (1.5) | 81 (2.1) |  |
| **Education level, n (%)** |  |  |  |
| No university degree | 4,825 (55.7) | 2,351 (62.0) |  |
| University degree | 3,841 (44.3) | 1,439 (38.0) |  |
| **Employment status, n (%)** |  |  |  |
| Unemployed | 206 (2.4) | 113 (3.0) |  |
| Part-time | 1,394 (16.1) | 648 (17.2) |  |
| Full-time | 1,560 (18.0) | 910 (24.2) |  |
| Home duties | 413 (4.8) | 220 (5.8) |  |
| Student | 10 (0.1) | 12 (0.3) |  |
| Retired | 5,075 (58.6) | 1,864 (49.5) |  |
| **Primary joint affected, n (%)** |  |  |  |
| Knee | 7,977 (80.3) | 3,933 (80.8) |  |
| Hip | 1,961 (19.7) | 932 (19.2) |  |
| **State/territory, n (%)** |  |  |  |
| Australian Capital Territory | 969 (9.8) | 340 (7.0) |  |
| New South Wales | 828 (8.3) | 405 (8.3) |  |
| Northern Territory | 59 (0.6) | 36 (0.7) |  |
| Queensland | 866 (8.7) | 441 (9.1) |  |
| South Australia | 1,419 (14.3) | 685 (14.1) |  |
| Tasmania | 470 (4.7) | 228 (4.7) |  |
| Victoria | 4,376 (44.1) | 2,367 (48.7) |  |
| Western Australia | 947 (9.5) | 361 (7.4) |  |
| **Clinic setting, n (%)** |  |  |  |
| Private | 8,296 (83.5) | 4,037 (83.0) |  |
| Public | 1,638 (16.5) | 826 (17.0) |  |
| **Program location*, n (%)** |  |  |  |
| Metropolitan | 7,331 (74.4) | 3,590 (74.8) |  |
| Rural and remote | 2,517 (25.6) | 1,212 (25.2) |  |
| **Knee measures, mean (SD), n** |  |  |  |
| KOOS-12 total (0-100) ^a^ | 50.9 (15.4), n = 7,973 | 48.0 (16.4), n = 3,909 | 2.9 (2.3, 3.5) |
| KOOS-12 pain (0-100) ^a^ | 52.6 (15.5), n = 6,971 | 50.4 (16.5), n = 3,096 | 2.2 (1.5, 2.9) |
| KOOS-12 function (0-100) ^a^ | 61.0 (18.7), n = 6,965 | 58.3 (19.7), n = 3,088 | 2.7 (1.9, 3.5) |
| KOOS-12 QoL (0-100) ^a^ | 42.7 (17.0), n = 7,968 | 40.8 (17.4), n = 3,902 | 1.9 (1.2, 2.5) |
| Knee worst pain VAS (0-100) ^b^ | 62.5 (23.3), n = 7,955 | 64.1 (23.1), n = 3,881 | -1.6 (-2.5, -0.7) |
| Knee average pain VAS (0-100) ^b^ | 44.4 (21.4), n = 7,941 | 47.6 (22.1), n = 3,853 | -3.2 (-4., -2.30) |
| **Hip measures, mean (SD), n** |  |  |  |
| HOOS-12 total (0-100) ^a^ | 53.4 (15.5), n = 1,960 | 50.0 (16.4), n = 927 | 3.4 (2.2, 4.7) |
| HOOS-12 pain (0-100) ^a^ | 52.7 (15.5), n = 1,697 | 51.1 (16.1), n = 697 | 1.6 (0.3, 3.0) |
| HOOS-12 function (0-100) ^a^ | 63.8 (18.3), n = 1,697 | 60.6 (18.9), n = 698 | 3.2 (1.6, 4.8) |
| HOOS-12 QoL (0-100) ^a^ | 46.9 (17.2), n = 1,960 | 44.3 (17.9), n = 926 | 2.5 (1.2, 3.9) |
| Hip worst pain VAS (0-100) ^b^ | 60.3 (23.6), n = 1,955 | 63.4 (23.6), n = 920 | -3.1 (-4.9, -1.2) |
| Hip average pain VAS (0-100) ^b^ | 42.8 (21.3), n = 1,954 | 47.1 (22.1), n = 915 | -4.3 (-6.0, -2.6) |
| **Combined measures** |  |  |  |
| EQ-5D-5L, utility score (0-1) ^c^ | 0.8 (0.1), n = 9,885 | 0.7 (0.2), n = 4,763 | 0.0 (0.0, 0.0) |
| EQ-5D-5L VAS (0-100) ^d^ | 73.6 (18.4), n = 9,879 | 70.8 (18.9), n = 4,747 | 2.8 (2.2, 3.4) |
| 30s chair stand test, count ^e^ | 11.1 (4.0), n = 9,738 | 10.9 (4.2), n = 4,517 | 0.2 (0.0, 0.3) |
| 40m walk test, m/s ^f^ | 1.5 (0.4), n = 8,960 | 1.5 (0.4), n = 4,044 | 0.0 (0.0, 0.0) |
| Abbreviations: EQ-5D-5L: European Quality of Life 5-Dimension 5-level; HOOS: Hip Disability and Osteoarthritis Outcome Score; KOOS: Knee Injury and Osteoarthritis Outcome Score; QoL: Quality of Life; n: Number; SD: Standard Deviation; VAS: Visual Analogue Scale.  *Remoteness measured using Modified Monash (MM) categories MM 1 to MM 7. Areas classified as metropolitan correspond to MM 1, which includes major cities characterized by high population densities and extensive infrastructure. Rural areas encompass MM 2 to MM 5, which include regional centres, large rural towns, medium rural towns, and small rural towns. Remote areas encompass MM 6 and MM 7, which consist of remote and very remote communities.  ^a^ Knee or hip-related pain, function, and quality of life are evaluated using KOOS/HOOS-12 question survey, scored from 0 (extreme  difficulty/pain) to 100 (no difficulty/pain).  ^b^ Assessed average and worst pain scores for affected joints over the past month, using a VAS from 0 (no pain) to 100 (worst pain).  ^c^ QoL measured across five EQ-5D-5L dimensions, scores from 0 (worst health) to 1 (best health).  ^d^ QoL measured using VAS, scored 0 (worst health) to 100 (best health)  ^e^ Physiotherapists assessed physical function using the 30-s chair-stand - patients were asked to stand up and sit down from the chair to perform as many repetitions as possible in 30 seconds.  ^f^ Physiotherapists assessed physical function/walking speed using a 40-meter walk test to measure the time (in seconds) taken to complete the 40-meter walk. | | | |

**eTable 4**. Baseline comparisons by clinic settings.

| Characteristics | Public clinic | Private clinic | Mean difference (95% CI) |
| --- | --- | --- | --- |
| Age (years), mean (SD), n | 67.4 (9.0), n = 1,584 | 66.3 (8.7), n = 8,192 | -1.1 (-1.6, -0.6) |
| Body mass index (kg/m^2^), mean (SD), n | 30.9 (7.3), n = 1,593 | 29.5 (6.3), n = 8,214 | -1.4 (-1.7, -1.0) |
| **Sex, n (%)** |  |  |  |
| Female | 1,097 (72.2) | 5,614 (72.3) |  |
| Male | 430 (28.2) | 2,156 (27.8) |  |
| **Marital Status, n (%)** |  |  |  |
| Single | 110 (7.7) | 392 (5.4) |  |
| Married/domestic partnership/de facto | 889 (62.1) | 5,408 (74.8) |  |
| Widowed | 139 (9.7) | 597 (8.3) |  |
| Divorced | 210 (14.7) | 609 (8.4) |  |
| Separated | 52 (3.6) | 133 (1.8) |  |
| Prefer not to say | 31 (2.2) | 96 (1.3) |  |
| **Education level, n (%)** |  |  |  |
| No university degree | 937 (65.5) | 3,888 (53.7) |  |
| University degree | 494 (34.5) | 3,347 (46.3) |  |
| **Employment status, n (%)** |  |  |  |
| Unemployed | 85 (6.0) | 121 (1.7) |  |
| Part-time | 203 (14.2) | 1,191 (16.5) |  |
| Full-time | 165 (11.6) | 1,395 (19.3) |  |
| Home duties | 90 (6.3) | 323 (4.5) |  |
| Student | 5 (0.4) | 5 (0.1) |  |
| Retired | 878 (61.6) | 4,197 (58.0) |  |
| **Primary joint affected, n (%)** |  |  |  |
| Knee | 1,331 (81.3) | 6,643 (80.1) |  |
| Hip | 307 (18.7) | 1,653 (19.9) |  |
| **State/territory, n (%)** |  |  |  |
| Australian Capital Territory | 373 (22.8) | 596 (7.2) |  |
| New South Wales | 42 (2.6) | 786 (9.5) |  |
| Northern Territory | 49 (3.0) | 10 (0.1) |  |
| Queensland | 207 (12.6) | 659 (7.9) |  |
| South Australia | 11 (0.7) | 1,408 (17.0) |  |
| Tasmania | 85 (5.2) | 385 (4.6) |  |
| Victoria | 850 (51.9) | 3,526 (42.5) |  |
| Western Australia | 21 (1.3) | 926 (11.2) |  |
| **Program location*, n (%)** |  |  |  |
| Metropolitan | 1,023 (65.9) | 6,308 (76.0) |  |
| Rural and remote | 529 (34.1) | 1,988 (24.0) |  |
| **Knee measures, mean (SD), n** |  |  |  |
| KOOS-12 total (0-100) ^a^ | 47.6 (16.5), n = 1,328 | 51.6 (15.1), n = 6,642 | 4.0 (3.1, 4.9) |
| KOOS-12 pain (0-100) ^a^ | 49.7 (16.4), n = 1,166 | 53.2 (15.3), n = 5,805 | 3.5 (2.5, 4.5) |
| KOOS-12 function (0-100) ^a^ | 56.1 (19.4), n = 1,166 | 62.0 (18.4), n = 5,799 | 5.8 (4.7, 7.0) |
| KOOS-12 QoL (0-100) ^a^ | 39.9 (18.1), n = 1,328 | 43.2 (16.7), n = 6,637 | 3.3 (2.3, 4.3) |
| Knee worst pain VAS (0-100) ^b^ | 63.8 (22.6), n = 1,325 | 62.2 (23.5), n = 6,627 | -1.6 (-3.0, -0.3) |
| Knee average pain VAS (0-100) ^b^ | 48.0 (21.9), n = 1,322 | 43.7 (21.3), n = 6,616 | -4.3 (-5.6, -3.1) |
| **Hip measures, mean (SD), n** |  |  |  |
| HOOS-12 total (0-100) ^a^ | 50.9 (17.2), n = 307 | 53.9 (15.1), n = 1,652 | 2.9 (1.1, 4.8) |
| HOOS-12 pain (0-100) ^a^ | 50.4 (17.1), n = 264 | 53.1 (15.1), n = 1,433 | 2.8 (0.7, 4.8) |
| HOOS-12 function (0-100) ^a^ | 60.0 (20.2), n = 264 | 64.5 (17.9), n = 1,433 | 4.5 (2.1, 6.9) |
| HOOS-12 QoL (0-100) ^a^ | 45.3 (18.5), n = 307 | 47.2 (16.9), n = 1,652 | 1.9 (-0.2, 3.9) |
| Hip worst pain VAS (0-100) ^b^ | 59.9 (24.3), n = 305 | 60.4 (23.5), n = 1,649 | 0.5 (-2.4, 3.4) |
| Hip average pain VAS (0-100) ^b^ | 44.9 (21.7), n = 305 | 42.4 (21.2), n = 1,648 | -2.5 (-5.1, 0.1) |
| **Combined measures** |  |  |  |
| EQ-5D-5L, utility score (0-1) ^c^ | 0.72 (0.18), n = 1,616 | 0.78 (0.14), n = 8,267 | 0.10 (0.00, 0.10) |
| EQ-5D-5L VAS (0-100) ^d^ | 69.6 (19.5), n = 1,609 | 74.4 (18.0), n = 8,268 | 4.8 (3.8, 5.8) |
| 30s chair stand test, count ^e^ | 10.5 (4.5), n = 1,577 | 11.2 (3.9), n = 8,157 | 0.6 (0.4, 0.9) |
| 40m walk test, m/s ^f^ | 1.4 (0.4), n = 1,411 | 1.5 (0.4), n = 7,546 | 0.0 (0.0, 0.0) |
| Abbreviations: EQ-5D-5L: European Quality of Life 5-Dimension 5-level; HOOS: Hip Disability and Osteoarthritis Outcome Score; KOOS: Knee Injury and Osteoarthritis Outcome Score; QoL: Quality of Life; n: Number; SD: Standard Deviation; VAS: Visual Analogue Scale.  *Remoteness measured using Modified Monash (MM) categories MM 1 to MM 7. Areas classified as "Metro" correspond to MM 1, which includes major cities characterised by high population densities and extensive infrastructure. Rural areas encompass MM 2 to MM 5, which include regional centres, large rural towns, medium rural towns, and small rural towns. Remote areas encompass MM 6 and MM 7, which consist of remote and very remote communities.  ^a^ Knee or hip-related pain, function, and quality of life are evaluated using KOOS/HOOS-12 question survey, scored from 0 (extreme difficulty/pain) to 100 (no difficulty/pain).  ^b^ Assessed average and worst pain scores for affected joints over the past month, using a VAS from 0 (no pain) to 100 (worst pain).  ^c^ QoL measured across five EQ-5D-5L dimensions, scores from 0 (worst health) to 1 (best health).  ^d^ QoL measured using VAS, scored 0 (worst health) to 100 (best health)  ^e^ Physiotherapists assessed physical function using the 30-s chair-stand - patients were asked to stand up and sit down from the chair to perform as many repetitions as possible in 30 seconds.  ^f^ Physiotherapists assessed physical function/walking speed using a 40-meter walk test to measure the time (in seconds) taken to complete the 40-meter walk. | | | |

**eTable 5**. Baseline comparisons by geographical locations.

| Characteristics | Metropolitan | Rural/remote | Mean difference (95% CI) |
| --- | --- | --- | --- |
| Age (years), mean (SD), n | 66.5 (8.8), n = 7,212 | 66.4 (8.6), n = 2,478 | 0.1 (-0.3, 0.5) |
| Body mass index (kg/m^2^), mean (SD), n | 29.5 (6.4), n = 7,254 | 30.1 (6.4), n = 2,474 | -0.6 (-0.9, -0.3) |
| **Sex, n (%)** |  |  |  |
| Female | 4,876 (71.4) | 1,777 (74.8) |  |
| Male | 1,958 (28.6) | 600 (25.2) |  |
| **Marital Status, n (%)** |  |  |  |
| Single | 387 (6.1) | 111 (5.0) |  |
| Married/domestic partnership/de facto | 4,605 (72.3) | 1,647 (74.3) |  |
| Widowed | 549 (8.6) | 177 (8.0) |  |
| Divorced | 598 (9.4) | 203 (9.2) |  |
| Separated | 130 (2.0) | 54 (2.4) |  |
| Prefer not to say | 101 (1.6) | 26 (1.2) |  |
| **Education level, n (%)** |  |  |  |
| No university degree | 3,323 (52.2) | 1,436 (64.7) |  |
| University degree | 3,047 (47.8) | 782 (35.3) |  |
| **Employment status, n (%)** |  |  |  |
| Unemployed | 149 (2.3) | 47 (2.1) |  |
| Part-time | 1,020 (16.0) | 367 (16.6) |  |
| Full-time | 1,175 (18.5) | 367 (16.6) |  |
| Home duties | 299 (4.7) | 111 (5.0) |  |
| Student | 5 (0.1) | 5 (0.2) |  |
| Retired | 3,716 (58.4) | 1,319 (59.5) |  |
| **Primary joint affected, n (%)** |  |  |  |
| Knee | 5,921 (80.8) | 1,971 (78.3) |  |
| Hip | 1,410 (19.2) | 546 (21.7) |  |
| **State/territory, n (%)** |  |  |  |
| Australian Capital Territory | 969 (13.2) | - |  |
| New South Wales | 442 (6.0) | 386 (15.3) |  |
| Northern Territory | - | 59 (2.3) |  |
| Queensland | 716 (9.8) | 64 (2.5) |  |
| South Australia | 1,173 (16.0) | 246 (9.8) |  |
| Tasmania | - | 470 (18.7) |  |
| Victoria | 3,202 (43.7) | 1,174 (46.6) |  |
| Western Australia | 829 (11.3) | 118 (4.7) |  |
| **Knee measures, mean (SD), n** |  |  |  |
| KOOS-12 total (0-100) ^a^ | 51.1 (15.2), n = 5,918 | 50.7 (16.0), n = 1,971 | 0.3 (-0.5, 1.1) |
| KOOS-12 pain (0-100) ^a^ | 52.9 (15.3), n = 5,153 | 52.0 (16.0), n = 1,744 | 1.0 (0.1, 1.8) |
| KOOS-12 function (0-100) ^a^ | 61.4 (18.5), n = 5,150 | 60.3 (19.0), n = 1,741 | 1.1 (0.1, 2.1) |
| KOOS-12 QoL (0-100) ^a^ | 42.6 (16.7), n = 5,915 | 43.1 (17.5), n = 1,969 | -0.4 (-1.3, 0.4) |
| Knee worst pain VAS (0-100) ^b^ | 62.3 (23.1), n = 5,904 | 62.5 (24.1), n = 1,966 | -0.2 (-1.3, 1.0) |
| Knee average pain VAS (0-100) ^b^ | 44.1 (21.2), n = 5,891 | 44.8 (21.9), n = 1,965 | -0.7 (-1.8, 0.4) |
| **Hip measures, mean (SD), n** |  |  |  |
| HOOS-12 total (0-100) ^a^ | 53.6 (15.4), n = 1,409 | 52.8 (15.7), n = 546 | 0.8 (-0.7, 2.3) |
| HOOS-12 pain (0-100) ^a^ | 53.1 (15.3), n = 1,216 | 51.6 (15.7), n = 477 | 1.6 (0.0, 3.2) |
| HOOS-12 function (0-100) ^a^ | 64.2 (18.3), n = 1,216 | 62.9 (18.4), n = 477 | 1.2 (-0.7, 3.2) |
| HOOS-12 QoL (0-100) ^a^ | 47.0 (16.9), n = 1,409 | 46.5 (17.9), n = 546 | 0.5 (-1.2, 2.2) |
| Hip worst pain VAS (0-100) ^b^ | 60.3 (23.4), n = 1,407 | 60.3 (24.4), n = 543 | 0.1 (-2.3, 2.4) |
| Hip average pain VAS (0-100) ^b^ | 42.6 (21.3), n = 1,405 | 43.4 (21.3), n = 544 | -0.8 (-2.9, 1.3) |
| **Combined measures** |  |  |  |
| EQ-5D-5L, utility score (0-1) ^c^ | 0.77 (0.15), n = 7,299 | 0.76 (0.15), n = 2,500 | 0.00 (0.00, 0.00) |
| EQ-5D-5L VAS (0-100) ^d^ | 73.7 (18.2), n = 7,294 | 73.6 (18.9), n = 2,500 | 0.1 (-0.7, 0.9) |
| 30s chair stand test, count ^e^ | 11.0 (4.0), n = 7,190 | 11.2 (4.1), n = 2,470 | -0.1 (-0.3, 0.0) |
| 40m walk test, m/s ^f^ | 1.5 (0.4), n = 6,647 | 1.5 (0.4), n = 2,244 | 0.0 (0.0, 0.0) |
| Abbreviations: EQ-5D-5L: European Quality of Life 5-Dimension 5-level; HOOS: Hip Disability and Osteoarthritis Outcome Score; KOOS: Knee Injury and Osteoarthritis Outcome Score; QoL: Quality of Life; n: Number; SD: Standard Deviation; VAS: Visual Analogue Scale.  *Remoteness measured using Modified Monash (MM) categories MM 1 to MM 7. Areas classified as "Metro" correspond to MM 1, which includes major cities characterized by high population densities and extensive infrastructure. Rural areas encompass MM 2 to MM 5, which include regional centres, large rural towns, medium rural towns, and small rural towns. Remote areas encompass MM 6 and MM 7, which consist of remote and very remote communities.  ^a^ Knee or hip-related pain, function, and quality of life are evaluated using KOOS/HOOS-12 question survey, scored from 0 (extreme difficulty/pain) to 100 (no difficulty/pain).  ^b^ Assessed average and worst pain scores for affected joints over the past month, using a VAS from 0 (no pain) to 100 (worst pain).  ^c^ QoL measured across five EQ-5D-5L dimensions, scores from 0 (worst health) to 1 (best health).  ^d^ QoL measured using VAS, scored 0 (worst health) to 100 (best health)  ^e^ Physiotherapists assessed physical function using the 30-s chair-stand - patients were asked to stand up and sit down from the chair to perform as many repetitions as possible in 30 seconds.  ^f^ Physiotherapists assessed physical function/walking speed using a 40-meter walk test to measure the time (in seconds) taken to complete the 40-meter walk. | | | |

**eTable 6**. Baseline comparisons by level of education.

| Characteristics | No university degree | University degree | Mean difference (95% CI) |
| --- | --- | --- | --- |
| Age (years), mean (SD), n | 66.9 (8.8), n = 4,799 | 66.4 (8.8), n = 3,831 | -0.5 (-0.9, -0.1) |
| Body mass index (kg/m^2^), mean (SD), n | 30.3 (6.5), n = 4,759 | 29.0 (6.4), n = 3,814 | -1.2 (-1.5, -0.9) |
| **Sex, n (%)** |  |  |  |
| Female | 3,487 (72.3) | 2,750 (71.7) |  |
| Male | 1,336 (27.7) | 1,087 (28.3) |  |
| **Marital Status, n (%)** |  |  |  |
| Single | 241 (5.0) | 261 (6.8) |  |
| Married/domestic partnership/de facto | 3,487 (72.3) | 2,808 (73.1) |  |
| Widowed | 482 (10.0) | 254 (6.6) |  |
| Divorced | 452 (9.4) | 367 (9.6) |  |
| Separated | 110 (2.3) | 75 (2.0) |  |
| Prefer not to say | 51 (1.1) | 76 (2.0) |  |
| **Employment status, n (%)** |  |  |  |
| Unemployed | 145 (3.0) | 61 (1.6) |  |
| Part-time | 713 (14.8) | 680 (17.7) |  |
| Full-time | 830 (17.2) | 729 (19.0) |  |
| Home duties | 309 (6.4) | 104 (2.7) |  |
| Student | 7 (0.2) | 3 (0.1) |  |
| Retired | 2,815 (58.4) | 2,260 (58.9) |  |
| **Primary joint affected, n (%)** |  |  |  |
| Knee | 3,882 (80.5) | 3,086 (80.3) |  |
| Hip | 943 (19.5) | 755 (19.7) |  |
| **State/territory, n (%)** |  |  |  |
| Australian Capital Territory | 411 (8.5) | 517 (13.5) |  |
| New South Wales | 408 (8.5) | 340 (8.9) |  |
| Northern Territory | 33 (0.7) | 5 (0.1) |  |
| Queensland | 469 (9.7) | 302 (7.9) |  |
| South Australia | 717 (14.9) | 535 (13.9) |  |
| Tasmania | 225 (4.7) | 195 (5.1) |  |
| Victoria | 2,002 (41.5) | 1,615 (42.1) |  |
| Western Australia | 560 (11.6) | 332 (8.6) |  |
| **Clinic setting, n (%)** |  |  |  |
| Private | 3,888 (80.6) | 3,347 (87.1) |  |
| Public | 937 (19.4) | 494 (12.9) |  |
| **Program location*, n (%)** |  |  |  |
| Metropolitan | 3,323 (69.8) | 3,047 (79.6) |  |
| Rural and remote | 1,436 (30.2) | 782 (20.4) |  |
| **Knee measures, mean (SD), n** |  |  |  |
| KOOS-12 total (0-100) ^a^ | 49.9 (15.3), n = 3,881 | 54.9 (14.4), n = 3,086 | 5.0 (4.3, 5.7) |
| KOOS-12 pain (0-100) ^a^ | 50.2 (15.6), n = 3,881 | 55.7 (14.9), n = 3,086 | 5.6 (4.8, 6.3) |
| KOOS-12 function (0-100) ^a^ | 58.1 (18.8), n = 3,879 | 64.6 (17.8), n = 3,084 | 6.5 (5.6, 7.3) |
| KOOS-12 QoL (0-100) ^a^ | 41.4 (17.7), n = 3,879 | 44.3 (16.5), n = 3,084 | 3.0 (2.1, 3.8) |
| Knee worst pain VAS (0-100) ^b^ | 65.0 (22.5), n = 3,871 | 60.0 (23.7), n = 3,081 | -5.0 (-6.1, -4.0) |
| Knee average pain VAS (0-100) ^b^ | 48.1 (21.3), n = 3,864 | 40.6 (21.0), n = 3,075 | -7.5 (-8.5, -6.5) |
| **Hip measures, mean (SD), n** |  |  |  |
| HOOS-12 total (0-100) ^a^ | 53.6 (15.5), n = 943 | 55.6 (14.7), n = 754 | 2.0 (0.6, 3.5) |
| HOOS-12 pain (0-100) ^a^ | 51.6 (15.7), n = 943 | 54.0 (15.1), n = 754 | 2.4 (0.9, 3.9) |
| HOOS-12 function (0-100) ^a^ | 62.1 (18.5), n = 943 | 66.0 (17.9), n = 754 | 3.9 (2.1, 5.6) |
| HOOS-12 QoL (0-100) ^a^ | 47.0 (17.8), n = 943 | 46.9 (16.7), n = 754 | -0.2 (-1.8, 1.5) |
| Hip worst pain VAS (0-100) ^b^ | 60.9 (23.2), n = 940 | 60.4 (23.9), n = 752 | -0.5 (-2.7, 1.8) |
| Hip average pain VAS (0-100) ^b^ | 44.9 (21.3), n = 939 | 41.1 (21.1), n = 752 | -3.9 (-5.9, -1.9) |
| **Combined measures** |  |  |  |
| EQ-5D-5L, utility score (0-1) ^c^ | 0.76 (0.16), n = 4,802 | 0.78 (0.14), n = 3,830 | 0.02 (0.02, 0.03) |
| EQ-5D-5L VAS (0-100) ^d^ | 73.5 (18.6), n = 4,803 | 73.9 (18.0), n = 3,830 | 0.4 (-0**.**4, 1.2) |
| 30s chair stand test, count ^e^ | 10.9 (4.1), n = 4,738 | 11.5 (4.1), n = 3,796 | 0.7 (0.5, 0.8) |
| 40m walk test, m/s ^f^ | 1.4 (0.4), n = 4,298 | 1.5 (0.4), n = 3,487 | 0.1 (0.1, 0.1) |
| Abbreviations: EQ-5D-5L: European Quality of Life 5-Dimension 5-level; HOOS: Hip Disability and Osteoarthritis Outcome Score; KOOS: Knee Injury and Osteoarthritis Outcome Score; QoL: Quality of Life; n: Number; SD: Standard Deviation; VAS: Visual Analogue Scale.  *Remoteness measured using Modified Monash (MM) categories MM 1 to MM 7. Areas classified as "Metro" correspond to MM 1, which includes major cities characterized by high population densities and extensive infrastructure. Rural areas encompass MM 2 to MM 5, which include regional centres, large rural towns, medium rural towns, and small rural towns. Remote areas encompass MM 6 and MM 7, which consist of remote and very remote communities.  ^a^ Knee or hip-related pain, function, and quality of life are evaluated using KOOS/HOOS-12 question survey, scored from 0 (extreme difficulty/pain) to 100 (no difficulty/pain).  ^b^ Assessed average and worst pain scores for affected joints over the past month, using a VAS from 0 (no pain) to 100 (worst pain).  ^c^ QoL measured across five EQ-5D-5L dimensions, scores from 0 (worst health) to 1 (best health).  ^d^ QoL measured using VAS, scored 0 (worst health) to 100 (best health)  ^e^ Physiotherapists assessed physical function using the 30-s chair-stand - patients were asked to stand up and sit down from the chair to perform as many repetitions as possible in 30 seconds.  ^f^ Physiotherapists assessed physical function/walking speed using a 40-meter walk test to measure the time (in seconds) taken to complete the 40-meter walk. | | | |

**eTable 7.** Change in study outcomes between public and private clinics over 3 months after imputations for missing data

| Study outcomes | Public,  Mean (95% CI) | | | Private,  Mean (95% CI) | | | Private vs public difference in change, mean (95% CI) * | P value |
| --- | --- | --- | --- | --- | --- | --- | --- | --- |
|  | Baseline | 3 months | Change at 3 months | Baseline | 3 months | Change at 3 months |  |  |
| **Knee measures** |  |  |  |  |  |  |  |  |
| KOOS-12 total (0-100) ^a^ | 46.5 (45.8, 47.2) | 56.5 (55.5, 57.5) | 10.0 (8.8, 11.2) | 50.6 (50.3, 51.0) | 62.1 (61.7, 62.5) | 11.5 (11.0, 12.0) | 1.4 (0.2, 2.7) | 0.023 |
| KOOS-12 pain (0-100) ^a^ | 48.5 (47.7, 49.3) | 57.9 (56.8, 59.0) | 9.4 (8.1, 10.7) | 52.6 (52.3, 53.0) | 63.5 (63.1, 63.9) | 10.9 (10.3, 11.4) | 1.5 (0.1, 2.8) | 0.032 |
| KOOS-12 function (0-100) ^a^ | 55.1 (54.2, 56.0) | 64.6 (63.4, 65.8) | 9.5 (8.0, 11.0) | 61.2 (60.8, 61.6) | 71.7 (71.2, 72.2) | 10.5 (9.8, 11.1) | 0.9 (-0.6, 2.5) | 0.230 |
| KOOS-12 QoL (0-100) ^a^ | 39.4 (38.6, 40.2) | 49.3 (48.3, 50.4) | 9.9 (8.6, 11.2) | 42.6 (42.3, 42.9) | 54.3 (53.8, 54.7) | 11.7 (11.1, 12.2) | 1.7 (0.4, 3.1) | 0.011 |
| Knee worst pain VAS (0-100) ^b^ | 64.4 (63.4, 65.3) | 51.9 (50.4, 53.4) | -12.4 (-14.1, -10.7) | 62.7 (62.2, 63.2) | 45.5 (44.9, 46.2) | -17.2 (-17.9, -16.4) | -4.7 (-6.6, -2.8) | **<0.001** |
| Knee average pain VAS (0-100) ^b^ | 49.1 (48.2, 50.1) | 37.9 (36.5, 39.2) | -11.3 (-12.9, -9.7) | 44.7 (44.2, 45.1) | 31.4 (30.8, 31.9) | -13.7 (-14.0, -12.6) | -2.0 (-3.7, -0.3) | 0.020 |
| **Hip measures** |  |  |  |  |  |  |  |  |
| HOOS-12 total (0-100) ^a^ | 49.3 (47.7, 50.8) | 57.7 (55.6, 59.8) | 8.4 (5.9, 11.0) | 52.9 (52.2, 53.5) | 61.3 (60.5, 62.2) | 8.5 (7.5, 9.5) | 0.0 (-2.6, 2.6) | 0.972 |
| HOOS-12 pain (0-100) ^a^ | 48.9 (47.2, 50.6) | 58.0 (55.6, 60.3) | 9.0 (6.2, 11.8) | 52.8 (52.2, 53.5) | 61.3 (60.4, 62.2) | 8.5 (7.4, 9.6) | -0.6 (-3.4, 2.3) | 0.695 |
| HOOS-12 function (0-100) ^a^ | 58.2 (56.3, 60.2) | 67.2 (64.7, 69.6) | 9.0 (5.8, 12.1) | 63.8 (63.0, 64.5) | 71.7 (70.7, 72.7) | 8.0 (6.7, 9.2) | -1.0 (-4.2, 2.2) | 0.547 |
| HOOS-12 QoL (0-100) ^a^ | 43.7 (42.0, 45.4) | 51.5 (49.3, 53.7) | 7.8 (5.1, 10.5) | 46.5 (45.8, 47.2) | 54.3 (53.4, 55.2) | 7.8 (6.7, 9.0) | 0.0 (-2.8, 2.9) | 0.984 |
| Hip worst pain VAS (0-100) ^b^ | 62.1 (59.9, 64.3) | 50.5 (47.5, 53.5) | -11.6 (-15.2, -8.0) | 61.2 (60.2, 62.1) | 48.6 (47.3, 49.9) | -12.6 (-14.2, -11.0) | -1.0 (-5.0, 3.0) | 0.625 |
| Hip average pain VAS (0-100) ^b^ | 47.4 (45.4, 49.4) | 37.0 (34.4, 39.6) | -10.4 (-13.6, -7.1) | 43.6 (42.7, 44.5) | 33.4 (32.3, 34.5) | -10.2 (-11.6, -8.8) | 0.2 (-3.3, 3.7) | 0.930 |
| **Combined measures** |  |  |  |  |  |  |  |  |
| EQ-5D-5L, utility score (0-1) ^c^ | 0.71 (0.70, 0.71) | 0.76 (0.75, 0.77) | 0.05 (0.04, 0.07) | 0.77 (-) | 0.82 (-) | 0.05 (-) | 0.00 (-0.01, 0.01) | 0.479 |
| EQ-5D-5L VAS (0-100) ^d^ | 68.9 (68.1, 69.7) | 72.4 (71.4, 73.3) | 3.5 (2.2, 4.7) | 73.5 (73.2, 73.8) | 76.9 (76.5, 77.3) | 3.4 (2.9, 4.0) | 0.0 (-1.3, 1.3) | 0.959 |
| 30s chair stand test, count ^e^ | 10.4 (10.3, 10.6) | 13.2 (12.9, 13.5) | 2.7 (2.4, 3.1) | 11.1 (11.1, 11.2) | 14.2 (14.0, 14.3) | 3.0 (2.9, 3.2) | 0.3 (-0.1, 0.6) | 0.098 |
| 40m walk test, m/s ^f^ | 1.36 (1.34, 1.38) | 1.56 (1.54, 1.59) | 0.21 (0.17, 0.24) | 1.49 (1.48, 1.50) | 1.68 (1.67, 1.70) | 0.19 (0.18, 0.21) | -0.01 (-0.05, 0 .02) | 0.482 |
| Abbreviations: CI: Confidence Interval; EQ-5D-5L: European Quality of Life 5-Dimension 5-level; HOOS: Hip Disability and Osteoarthritis Outcome Score; KOOS: Knee Injury and Osteoarthritis Outcome Score; n: Number; QoL: Quality of Life; VAS: Visual Analogue Scale.  *The mean difference is calculated as private minus public. The change was estimated using linear mixed-effect model. No missing values were imputed.  ^a^ Knee or hip-related pain, function, and quality of life are evaluated using KOOS/HOOS-12 question survey, scored from 0 (extreme  difficulty/pain) to 100 (no difficulty/pain).  ^b^ Assessed average and worst pain scores for affected joints over the past month, using a VAS from 0 (no pain) to 100 (worst pain).  ^c^ QoL measured across five EQ-5D-5L dimensions, scores from 0 (worst health) to 1 (best health).  ^d^ QoL measured using VAS, scored from 0 (worst health) to 100 (best health)  ^e^ Physiotherapists assessed physical function using the 30-s chair-stand - patients were asked to stand up and sit down from the chair to perform as many repetitions as possible in 30 seconds.  ^f^ Physiotherapists assessed physical function/walking speed using a 40-meter walk test to measure the time (in seconds) taken to complete the 40-meter walk. | | | | | | | | |

**eTable 8**. Change in study outcomes between metropolitan and rural/remote locations over 3 months after imputations for missing data

| Study outcomes | Metropolitan,  Mean (95% CI) | | | Rural and remote,  Mean (95% CI) | | | Metropolitan vs rural and remote difference in change, mean (95% CI) * | p value |
| --- | --- | --- | --- | --- | --- | --- | --- | --- |
|  | Baseline | 3 months | Change at 3 months | Baseline | 3 months | Change at 3 months |  |  |
| **Knee measures** |  |  |  |  |  |  |  |  |
| KOOS-12 total (0-100) ^a^ | 50.2 (49.8, 50.5) | 61.4 (61.0, 61.8) | 11.2 (10.7, 11.8) | 49.7 (49.1, 50.3) | 61.0 (60.2, 61.8) | 11.3 (10.4, 12.3) | -0.1 (-1.2, 1.0) | 0.852 |
| KOOS-12 pain (0-100) ^a^ | 52.4 (52.1, 52.8) | 62.8 (62.4, 63.3) | 10.4 (9.8, 11.0) | 51.0 (50.3, 51.6) | 62.2 (61.4, 63.1) | 11.3 (10.2, 12.3) | -0.8 (-2.0, 0.3) | 0.166 |
| KOOS-12 function (0-100) ^a^ | 60.8 (60.3, 61.2) | 70.9 (70.4, 71.5) | 10.2 (9.5, 10.9) | 59.0 (58.3, 59.8) | 69.8 (68.9, 70.7) | 10.8 (9.6, 12.0) | -0.6 (-2.0, 0.7) | 0.376 |
| KOOS-12 QoL (0-100) ^a^ | 42.1 (41.7, 42.4) | 53.6 (53.1, 54.0) | 11.5 (10.9, 12.1) | 42.4 (41.7, 43.0) | 53.5 (52.7, 54.4) | 11.2 (10.1, 12.2) | 0.3 (-0.8, 1.5) | 0.578 |
| Knee worst pain VAS (0-100) ^b^ | 62.8 (62.3, 63.3) | 46.4 (45.7, 47.1) | -16.4 (-17.2, -15.6) | 63.3 (62.4, 64.2) | 46.7 (45.5, 47.9) | -16.6 (-18.0, -15.1) | 0.2 (-1.5, 1.8) | 0.828 |
| Knee average pain VAS (0-100) ^b^ | 45.1 (44.6, 45.5) | 32.2 (31.6, 32.8) | -12.9 (-13.6, -12.2) | 46.1 (45.3, 46.9) | 32.7 (31.7, 33.8) | -13.4 (-14.7, -12.1) | 0.5 (-1.0, 1.9) | 0.521 |
| **Hip measures** |  |  |  |  |  |  |  |  |
| HOOS-12 total (0-100) ^a^ | 52.6 (51.9, 53.3) | 60.8 (59.9, 61.7) | 8.2 (7.1, 9.4) | 51.6 (50.5, 52.7) | 60.6 (59.1, 62.1) | 9.0 (7.2, 10.8) | -0.8 (-2.9, 1.4) | 0.483 |
| HOOS-12 pain (0-100) ^a^ | 52.8 (52.0, 53.5) | 60.9 (60.0, 61.9) | 8.2 (6.9, 9.4) | 50.8 (49.7, 52.0) | 60.2 (58.6, 61.8) | 9.4 (7.5, 11.3) | -1.2 (-3.5, 1.1) | 0.291 |
| HOOS-12 function (0-100) ^a^ | 63.4 (62.5, 64.3) | 71.3 (70.3, 72.4) | 7.9 (6.5, 9.3) | 61.5 (60.2, 62.9) | 70.1 (68.3, 71.8) | 8.5 (6.3, 10.7) | -0.6 (-3.2, 2.0) | 0.665 |
| HOOS-12 QoL (0-100) ^a^ | 46.2 (45.4, 46.9) | 53.9 (52.9, 54.8) | 7.7 (6.5, 8.9) | 45.8 (44.5, 47.0) | 53.8 (52.2, 55.5) | 8.1 (6.1, 10.1) | -0.4 (-2.7, 2.0) | 0.755 |
| Hip worst pain VAS (0-100) ^b^ | 61.1 (60.1, 62.1) | 48.7 (47.3, 50.1) | -12.4 (-14.1, -10.7) | 61.7 (60.0, 63.4) | 49.5 (47.2, 51.8) | -12.2 (-15.0, -9.5) | -0.2 (-3.4, 3.0) | 0.905 |
| Hip average pain VAS (0-100) ^b^ | 43.9 (42.9, 44.8) | 33.4 (32.2, 34.6) | -10.5 (-12.0, -9.0) | 45.0 (43.5, 46.4) | 35.5 (33.5, 37.4) | -9.5 (-11.9, -7.1) | -1.0 (-3.8, 1.9) | 0.505 |
| **Combined measures** |  |  |  |  |  |  |  |  |
| EQ-5D-5L, utility score (0-1) ^c^ | 0.76 (0.76, 0.77) | 0.81 (0.81, 0.82) | 0.05 (-) | 0.76 (0.75, 0.76) | 0.81 (0.80, 0.81) | 0.05 (0.04, 0.06) | 0.00(-0.01, 0.01) | 0.889 |
| EQ-5D-5L VAS (0-100) ^d^ | 72.9 (72.5, 73.2) | 76.2 (75.7, 76.6) | 3.3 (2.7, 3.8) | 72.5 (71.9, 73.1) | 76.4 (75.6, 77.1) | 3.8 (2.9, 4.8) | -0.5 (-1.7, 0.6) | 0.329 |
| 30s chair stand test, count ^e^ | 11.0 (11.0, 11.1) | 14.0 (13.9, 14.1) | 3.0 (2.8, 3.1) | 11.0 (10.9, 11.2) | 14.0 (13.8, 14.2) | 3.0 (2.7, 3.2) | 0.0 (-0.3, 0.3) | 0.982 |
| 40m walk test, m/s ^f^ | 1.48 (1.47, 1.49) | 1.68 (1.66, 1.69) | 0.20 (0.18, 0.21) | 1.45 (1.44, 1.46) | 1.64 (1.62, 1.66) | 0.19 (0.16, 0.21) | 0.01 (-0.02, 0.04) | 0.485 |
| Abbreviations: CI: Confidence Interval; EQ-5D-5L: 5-Level: Euroqol-5-Dimensional Version; HOOS: Hip Disability and Osteoarthritis Outcome Score; KOOS: Knee Injury and Osteoarthritis Outcome Score; n: Number; QoL: Quality of Life; VAS: Visual Analogue Scale.  *The mean difference calculated as metropolitan minus rural/remote. The change was estimated using linear mixed-effect model. No missing values were imputed.  ^a^ Knee or hip-related pain, function, and quality of life are evaluated using KOOS/HOOS-12 question survey, scored from 0 (extreme  difficulty/pain) to 100 (no difficulty/pain).  ^b^ Assessed average and worst pain scores for affected joints over the past month, using a VAS from 0 (no pain) to 100 (worst pain).  ^c^ QoL measured across five EQ-5D-5L dimensions, scores from 0 (worst health) to 1 (best health).  ^d^ QoL measured using VAS, scored 0 (worst health) to 100 (best health)  ^e^ Physiotherapists assessed physical function using the 30-s chair-stand - patients were asked to stand up and sit down from the chair to perform as many repetitions as possible in 30 seconds.  ^f^ Physiotherapists assessed physical function/walking speed using a 40-meter walk test to measure the time (in seconds) taken to complete the 40-meter walk. | | | | | | | | |

**eTable 9**. Change in study outcomes based on highest level of education over 3 months after imputations for missing data.

| Study outcomes | No university degree Mean (95% CI) | | | University degree Mean (95% CI) | | | University degree vs no university degree in change  mean (95% CI) * | P value |
| --- | --- | --- | --- | --- | --- | --- | --- | --- |
|  | Baseline | 3 months | Change at 3 months | Baseline | 3 months | Change at 3 months |  |  |
| **Knee measures** |  |  |  |  |  |  |  |  |
| KOOS-12 total (0-100) ^a^ | 49.3 (48.9, 49.7) | 59.9 (59.3, 60.4) | 10.5 (9.9, 11.2) | 54.3 (53.8, 54.7) | 64.9 (64.4, 65.5) | 10.7 (10.0, 11.4) | 0.1 (-0.9, 1.1) | 0.835 |
| KOOS-12 pain (0-100) ^a^ | 49.6 (49.2, 50.0) | 60.2 (59.6, 60.8) | 10.6 (9.9, 11.2) | 55.1 (54.7, 55.6) | 65.6 (65.0, 66.2) | 10.4 (9.7, 11.2) | -0.1 (-1.1, 0.9) | 0.822 |
| KOOS-12 function (0-100) ^a^ | 57.5 (57.0, 58.0) | 67.8 (67.2, 68.4) | 10.3 (9.5, 11.1) | 63.9 (63.3, 64.4) | 73.9 (73.3, 74.6) | 10.1 (9.2, 10.9) | -0.3 (-1.4, 0.9) | 0.669 |
| KOOS-12 QoL (0-100) ^a^ | 40.8 (40.4, 41.3) | 51.7 (51.1, 52.3) | 10.9 (10.1, 11.6) | 43.8 (43.3, 44.3) | 55.3 (54.6, 55.9) | 11.4 (10.7, 12.2) | 0.6 (-0.5, 1.7) | 0.297 |
| Knee worst pain VAS (0-100) ^b^ | 65.2 (64.7, 65.8) | 50.2 (49.3, 51.0) | -15.1 (-16.1, -14.1) | 60.8 (60.1, 61.5) | 44.3 (43.4, 45.2) | -16.5 (-17.6, -15.3) | -1.4 (-2.9, 0.1) | 0.070 |
| Knee average pain VAS (0-100) ^b^ | 49.0 (48.4, 49.5) | 36.7 (35.9, 37.4) | -12.3 (-13.2, -11.4) | 41.8 (41.1, 42.4) | 29.0 (28.2, 29.7) | -12.8 (-13.8, -11.8) | -0.5 (-1.9, 0.9) | 0.464 |
| **Hip measures** |  |  |  |  |  |  |  |  |
| HOOS-12 total (0-100) ^a^ | 52.7 (51.8, 53.5) | 61.2 (60.1, 62.4) | 8.6 (7.2, 9.9) | 55.3 (54.4, 56.2) | 62.8 (61.6, 64.0) | 7.5 (6.0, 8.9) | -1.1 (-3.1, 0.9) | 0.293 |
| HOOS-12 pain (0-100) ^a^ | 50.9 (50.1, 51.7) | 60.1 (59.0, 61.3) | 9.2 (7.8, 10.6) | 54.0 (53.0, 54.9) | 61.6 (60.4, 62.9) | 7.6 (6.1, 9.2) | -1.6 (-0.5, 3.7) | 0.130 |
| HOOS-12 function (0-100) ^a^ | 60.9 (59.9, 61.9) | 69.7 (68.5, 71.0) | 8.8 (7.2, 10.4) | 65.5 (64.4, 66.6) | 72.7 (71.3, 74.0) | 7.2 (5.4, 8.9) | -1.6 (-4.0, 0.7) | 0.171 |
| HOOS-12 QoL (0-100) ^a^ | 46.2 (45.2, 47.1) | 53.8 (52.6, 55.1) | 7.7 (6.1, 9.2) | 46.4 (45.4, 47.5) | 54.1 (52.8, 55.5) | 7.7 (6.0, 9.3) | 0.0 (-2.3, 2.3) | 0.993 |
| Hip worst pain VAS (0-100) ^b^ | 62.3 (61.1, 63.6) | 50.2 (48.4, 51.9) | -11.4 (-13.7, -9.0) | 60.5 (59.1, 62.0) | 49.2 (47.3, 51.1) | -12.2 (-14.2, -10.1) | -0.8 (-2.3, 4.0) | 0.600 |
| Hip average pain VAS (0-100) ^b^ | 46.8 (45.7, 48.0) | 36.2 (34.8, 37.7) | -10.6 (-12.4, -8.8) | 41.6 (40.3, 42.9) | 32.6 (31.0, 34.2) | -9.0 (-11.1, -7.0) | 1.6 (-1.2, 4.3) | 0.261 |
| **Combined measures** |  |  |  |  |  |  |  |  |
| EQ-5D-5L, utility score (0-1) ^c^ | 0.75 (-) | 0.80 (-) | 0.05 (0.04, 0.06) | 0.78 (0.77, 0.78) | 0.82 (0.82, 0.83) | 0.04 (0.04, 0.05) | -0.01 (-0.01, 0.00) | 0.203 |
| EQ-5D-5L VAS (0-100) ^d^ | 72.4 (72.0, 72.8) | 75.6 (75.0, 76.1) | 3.2 (2.5, 3.9) | 73.3 (72.8, 73.8) | 76.5 (75.9, 77.1) | 3.2 (2.5, 4.0) | 0.0 (-1.0, 1.1) | 0.971 |
| 30s chair stand test, count ^e^ | 10.8 (10.7, 10.9) | 13.8 (13.6, 13.9) | 2.9 (2.7, 3.1) | 11.5 (11.4, 11.6) | 14.5 (14.3, 14.7) | 3.0 (2.8, 3.2) | 0.1 (-0.2, 0.4) | 0.573 |
| 40m walk test, m/s ^f^ | 1.41 (1.40, 1.42) | 1.59 (1.58, 1.61) | 0.18 (0.16, 0.20) | 1.53 (1.52, 1.54) | 1.72 (1.70, 1.74) | 0.19 (0.17, 0.22) | 0.01 (-0.00, 0.02) | 0.437 |
| Abbreviations: CI: Confidence Interval; EQ-5D-5L: European Quality of Life 5-Dimension 5-level; HOOS: Hip Disability and Osteoarthritis Outcome Score; KOOS: Knee Injury and Osteoarthritis Outcome Score; n: Number; QoL: Quality of Life; VAS: Visual Analogue Scale.  *The mean difference is calculated as university degree minus no university degree. The change was estimated using linear mixed-effect model. No missing values were imputed.  ^a^ Knee or hip-related pain, function, and quality of life are evaluated using KOOS/HOOS-12 question survey, scored from 0 (extreme  difficulty/pain) to 100 (no difficulty/pain).  ^b^ Assessed average and worst pain scores for affected joints over the past month, using a VAS from 0 (no pain) to 100 (worst pain).  ^c^ QoL measured across five EQ-5D-5L dimensions, scores from 0 (worst health) to 1 (best health).  ^d^ QoL measured using VAS, scored 0 (worst health) to 100 (best health)  ^e^ Physiotherapists assessed physical function using the 30-s chair-stand - patients were asked to stand up and sit down from the chair to perform as many repetitions as possible in 30 seconds.  ^f^ Physiotherapists assessed physical function/walking speed using a 40-meter walk test to measure the time (in seconds) taken to complete the 40-meter walk. | | | | | | | | |
